# Multi-messenger gravitational lensing

**DOI:** 10.1098/rsta.2024.0134

**Published:** 2025-05-01

**Authors:** Graham P. Smith, Tessa Baker, Simon Birrer, Christine E. Collins, Jose Maria Ezquiaga, Srashti Goyal, Otto A. Hannuksela, Phurailatpam Hemanta, Martin A. Hendry, Justin Janquart, David Keitel, Andrew J. Levan, Rico K. L. Lo, Anupreeta More, Matt Nicholl, Inés Pastor-Marazuela, Andrés I. Ponte Pérez, Helena Ubach, Laura E. Uronen, Mick Wright, Miguel Zumalacarregui, Federica Bianco, Mesut Çalişkan, Juno C. L. Chan, Elena Colangeli, Benjamin P. Gompertz, Christopher P. Haines, Erin E. Hayes, Bin Hu, Gavin P. Lamb, Anna Liu, Soheb Mandhai, Harsh Narola, Quynh Lan Nguyen, Jason S. C. Poon, Dan Ryczanowski, Eungwang Seo, Anowar J. Shajib, Xikai Shan, Nial Tanvir, Luka Vujeva

**Affiliations:** ^1^ School of Physics and Astronomy, University of Birmingham, Edgbaston B15 2TT, UK; ^2^ Department of Astrophysics, University of Vienna, Türkenschanzstrasse 17, 1180 Vienna, Austria; ^3^ Institute of Cosmology and Gravitation, University of Portsmouth, Portsmouth PO1 3FX, UK; ^4^ Department of Physics and Astronomy, Stony Brook University, Stony Brook, NY 11794, USA; ^5^ School of Physics, Trinity College Dublin, The University of Dublin, Dublin 2, Ireland; ^6^ GSI Helmholtzzentrum, Schwerionenforschung, Planckstraße 1, 64291 Darmstadt, Germany; ^7^ Center of Gravity, Niels Bohr Institute, Blegdamsvej 17, 2100 Copenhagen, Denmark; ^8^ Max-Planck-Institute for Gravitational Physics (Albert Einstein Institute), Am Mühlenberg 1, Potsdam-Golm 14476, Germany; ^9^ Department of Physics, The Chinese University of Hong Kong, Shatin, Hong Kong; ^10^ SUPA, School of Physics and Astronomy, University of Glasgow, Glasgow G12 8QQ, UK; ^11^ Center for Cosmology, Particle Physics and Phenomenology-CP3, Université Catholique de Louvain, Louvain-la-Neuve B-1348, Belgium; ^12^ Royal Observatory of Belgium, Avenue Circulaire, 3, 1180 Uccle, Belgium; ^13^ Departament de Física, Universitat de les Illes Balears, IAC3–IEEC,Crta.Valldemossa km 7.5, E-07122 Palma, Spain; ^14^ Department of Astrophysics/IMAPP, Radboud Universiteit, Nijmegen, P.O. Box 9010, Nijmegen 6500 GL, The Netherlands; ^15^ Department of Physics, University of Warwick, Coventry CV4 7AL, UK; ^16^ Inter-University Centre for Astronomy and Astrophysics, Post Bag 4, Ganeshkhind, Pune 411007, India; ^17^ Kavli Institute for the Physics and Mathematics of the Universe (WPI), University of Tokyo, Kashiwa, Chiba 277-8583, Japan; ^18^ Astrophysics Research Centre, School of Mathematics and Physics, Queen's University Belfast, Belfast BT7 1NN, UK; ^19^ Jodrell Bank Centre for Astrophysics, University of Manchester, Oxford Road, Manchester M13 9PL, UK; ^20^ Institut de Ciènciesdel Cosmos (ICCUB), Universitat de Barcelona (UB), c. Martí i Franqués,1, 08028 Barcelona, Spain; ^21^ Departament de Física Quàntica i Astrofísica (FQA), Universitat de Barcelona (UB), c. Martí i Franqués, 1, 08028 Barcelona, Spain; ^22^ Department of Physics, Institute for Gravitational and Subatomic Physics (GRASP), Utrecht University, Princetonplein 1, 3584 CC Utrecht, The Netherlands; ^23^ Nikhef– National Institute for Subatomic Physics, Science Park, 1098 NG Amsterdam, The Netherlands; ^24^ University of Delaware, Department of Physics and Astronomy, 107 The Green, Newark, DE 19716, USA; ^25^ University of Delaware, Joseph R. Biden School of Public Policy, Graham Hall, 184 Academy Street, Newark, DE 19716, USA; ^26^ Vera C. Rubin Observatory, Tucson, AZ 85719, USA; ^27^ William H. Miller III Department of Physics and Astronomy, Johns Hopkins University, 3400 N Charles Street, Baltimore, MD 21218, USA; ^28^ Institute of Gravitational Wave Astronomy, University of Birmingham, Edgbaston B15 2TT, UK; ^29^ Instituto de Astronomía y Ciencias Planetarias de Atacama (INCT), Universidad de Atacama, Copayapu 485, Copiapó, Chile; ^30^ Institute of Astronomy and Kavli Institute for Cosmology, University of Cambridge, Madingley Road, Cambridge CB3 0HA, UK; ^31^ School of Physics and Astronomy, Beijing Normal University, Beijing 100875, People's Republic of China; ^32^ Astrophysics Research Institute, Liverpool John Moores University, IC2 Liverpool Science Park, 146 Brownlow Hill, Liverpool L3 5RF, UK; ^33^ Phenikaa Institute for Advanced Study, Phenikaa University, Hanoi 12116, Vietnam; ^34^ Department of Astronomy and Astrophysics, University of Chicago, Chicago, IL 60637, USA; ^35^ Kavli Institute for Cosmological Physics, University of Chicago, Chicago, IL 60637, USA; ^36^ Center for Astronomy, Space Science and Astrophysics, Independent University, Bangladesh, Dhaka 1229, Bangladesh; ^37^ NHFP Einstein Fellow; ^38^ Department of Astronomy, Tsinghua University, Beijing 100084, People's Republic of China; ^39^ School of Physics and Astronomy, University of Leicester, University Road, Leicester LE1 7RH, UK

**Keywords:** gravitational lensing, gravitational waves, kilonova, gamma-ray burst, time domain astronomy, multi-messenger astronomy

## Abstract

We introduce the rapidly emerging field of multi-messenger gravitational lensing—the discovery and science of gravitationally lensed phenomena in the distant universe through the combination of multiple messengers. This is framed by gravitational lensing phenomenology that has grown since the first discoveries in the twentieth century, messengers that span 30 orders of magnitude in energy from high-energy neutrinos to gravitational waves, and powerful ‘survey facilities’ that are capable of continually scanning the sky for transient and variable sources. Within this context, the main focus is on discoveries and science that are feasible in the next 5–10 years with current and imminent technology including the LIGO–Virgo–KAGRA network of gravitational wave detectors, the Vera C. Rubin Observatory and contemporaneous gamma/X-ray satellites and radio surveys. The scientific impact of even one multi-messenger gravitational lensing discovery will be transformational and reach across fundamental physics, cosmology and astrophysics. We describe these scientific opportunities and the key challenges along the path to achieving them. This article therefore describes the consensus that emerged at the eponymous Theo Murphy meeting in March 2024, and also serves as an introduction to this Theo Murphy meeting issue.

This article is part of the Theo Murphy meeting issue ‘Multi-messenger gravitational lensing (Part 2)’.

## Executive summary

In recent years, a broad consensus has developed that the multi-messenger discovery and science of gravitationally lensed phenomena in the distant universe is inevitable and will deliver scientific breakthroughs across some of the biggest open questions in fundamental physics, cosmology and astrophysics. Many of these questions are shared across the US Decadal Review, the AstroNet Roadmap and the science books of major facilities including the Vera C. Rubin Observatory (Rubin), Square Kilometre Array (SKA), next-generation gravitational wave (GW) detectors and 30 m class telescopes.

Multi-messenger gravitational lensing is well-placed to make decisive contributions on questions relating to the nature of gravity, the cosmological model including the expansion rate of the Universe and the nature of dark matter (DM), the demographics and formation channels of compact objects, the chemical enrichment of the Universe via the r-process, the equation of state (EoS) of dense nuclear matter, connections between and physics of diverse explosive transient populations, and the host galaxies of GW sources. Many of these are feasible within the next 5−10 years, i.e. on a timescale that is accelerated relative to that which is feasible without assistance from gravitational lensing.

These exciting opportunities are driven both by the discoveries of the last decade and by the rapid advances in detector sensitivity that together span ≃30 orders of magnitude in energy scale, including high-energy neutrinos, gamma/X-rays, optical and infrared (IR) photons, radio waves and GWs. In particular, the synergy between the superb arrival time precision of neutrino, gamma-ray, radio and GW detectors and the superb angular precision of optical/IR detectors and upcoming radio interferometers will drive the science in the coming decade and beyond.

Multi-messenger gravitational lensing advances in testing the nature of gravity will benefit from both the magnifying power of gravitational lenses to probe long travel times and multiple detections of the same source to boost the effective number of GW detectors. Generically, long travel times will significantly boost the sensitivity of searches for departures from General Relativity (GR), because potential deviations accumulate over large cosmological distances. Moreover, polarization constraints are the next frontier for tests of gravity with GWs. Therefore, multiple detections of the same chirp, due to gravitational lensing, will at least double the effective number of GW detectors that probe whether the number of GW polarization modes exceeds the two predicted from GR.

Multi-messenger gravitational lensing advances in cosmology will be driven by the complementary arrival time and angular position accuracies of the respective messengers, and the wave-like nature of the GW signals. A multi-messenger time-delay cosmography measurement of the Hubble Constant, $H_{0}$, will suppress the uncertainty on the arrival time difference measurement to a negligible level, will bring complementary insights into microlensing-related systematics, and for short arrival time differences may leverage the wave nature of GWs to break the mass-sheet degeneracy. There is also the exciting prospect of combining time-delay cosmography with standard siren cosmology in a single multi-messenger lensing cosmology experiment. On smaller scales, complementary constraints from different messengers, including their sensitivity to microlensing signatures, will deliver novel constraints on the DM subhalo mass function, stellar mass function and compact DM.

Multi-messenger gravitational magnification and arrival time differences will also open new windows on the physics of compact binary coalescences (CBCs; also referred to as binary compact object mergers) at high redshift. One of the biggest unsolved mysteries following the discovery of AT2017gfo, the kilonova counterpart to GRB170817A/GW170817, is the physical interpretation of the early blue kilonova emission. Very early rest-frame ultraviolet (UV) observations are crucial to break degeneracies between competing models. Multiple detections of the same gravitationally lensed kilonova can access this early phase of evolution, in a potentially spectacular fashion if the second image arrives while optical target of opportunity (ToO) observations are following up the first GW image that arrived. Detection of lensed gamma-ray burst (GRB) counterparts will further constrain the physics of gravitationally lensed CBCs that emit electromagnetic (EM) radiation, including experiments to probe the structure of GRB jets, benefiting from the different lines of sight to the jet afforded by gravitational lensing. Progenitor compact binary populations will also be probed, for example by testing objects that appear to populate relatively sparse regions of parameter space, such as the putative gap between neutron star (NS) and black hole (BH) masses, and the transition from the most massive stellar remnant BHs to intermediate-mass BHs. This progress, coupled with rapid progress in, e.g. fast radio bursts (FRBs), will also drive fresh insights into the putative association of some FRBs with CBCs.

This article concentrates on discoveries and science that are within reach in the next 5−10 years, with a broad focus on ground-based GW detectors, Rubin’s imminent Legacy Survey of Space and Time (LSST) and contemporaneous gamma/X-ray satellites and radio surveys. As such, the focus is on facilities that, in complementary ways, continually monitor the celestial sphere and/or are capable of rapid ToO observations in response to detections via other messengers. ‘Static sky’ discoveries are also of huge importance to multi-messenger gravitational lensing discoveries and science because the several orders of magnitude expansion of the census of gravitational lenses from Rubin/LSST, *Euclid* and their contemporaries will provide an unprecedented and comprehensive view of the high-magnification lines-of-sight to the distant universe.

Exciting scientific opportunities naturally come with challenges that the community must overcome, in this case on a timescale of 3−5 years. The most obvious cross-cutting challenge is to localize gravitationally lensed CBCs that are discovered via messengers with large localization uncertainties (GWs and GRBs) to ≲1arcsec, i.e. the angular scale of gravitational lensing and the gravitationally lensed host galaxies. This requires cross-community collaboration, including developing efficient methods to select candidates, plan follow-up observations and exploit synergies with rapidly growing gravitational lens catalogues. Example outcomes of cross-community collaboration include definitions of appropriate data-sharing requirements and protocols, and end-to-end simulations of multi-messenger gravitational lensing source populations, signals and detection strategies.

Robust multi-messenger gravitational lensing discoveries and interpretation of non-detections also require significant advances in our knowledge of the gravitational lens population in the observable universe. As alluded to above, the zeroth-order cross-cutting requirement is to build large and well-defined samples of gravitational lenses from EM surveys, with well-calibrated selection functions. However, robust multi-messenger gravitational lensing discovery strategies also require the internal structure of the lenses in these samples to be characterized as a function of the lens mass. Specifically, the density profile slope and density of lenses at their Einstein radii are the key parameters—in addition to Einstein radius—that control the expected arrival time difference and image separation for a given lens magnification.

Essential progress is also required in preparation for specific science cases including those sketched above, for example:

— incorporation of ultra-precise arrival time difference measurements into time-delay cosmology inference pipelines;
— detailed simulations of EM-bright CBCs with different mass ratios and NS equations of state;
— model-agnostic analysis pipelines for GW propagation, polarization and birefringence tests of GR;
— detailed theoretical predictions for how these phenomena present in cosmologically motivated theories of gravity beyond GR;
— the theory and computation of microlensing in the wave optics regime, models for data analysis and low-latency microlensing searches for EM follow-up;
— development of methods to identify and follow up candidate gravitationally lensed GRBs and FRBs in real-time, to enable discoveries before and during future GW runs;
— detailed simulations of gravitational lensing as a probe of GRB jet structure, as a path to optimize gravitationally lensed GRB searches.

## 1. Introduction

Multi-messenger gravitational lensing combines multiple messengers to discover and study transient and variable phenomena in gravitationally lensed host galaxies in the distant universe (typically redshifts of z≳1) to probe a broad range of physics. The messengers span at least 30 orders of magnitude in energy, from $≃10^{8}\;\mathrm{GeV}$ to $≃10^{-14}\;\mathrm{eV}$, and include high-energy neutrinos, gamma- and X-rays, UV/optical/IR photons, radio waves and GWs. As they traverse the gravitational potential of a dense foreground structure such as a galaxy or group/cluster of galaxies, several paths of least action through the potential may cause multiple ‘images’ of the source to arrive at an observer at different times. Each of the images corresponds to a different trajectory that is perturbed relative to that in the absence of lensing by up to ≃1arcmin, and their flux can be magnified significantly.

Messengers associated with transient and variable sources typically emanate from sources related to compact objects (black holes and neutron stars), some of which are the end points of stellar evolution. These sources include the collapse of stellar cores (supernovae, GRBs, neutrinos and GWs), CBCs (GWs, kilonovae, GRBs and their afterglows), phenomena associated with supermassive black holes in galaxies (active galactic nuclei including blazars, and tidal disruption of stars), plus fast-fading X-ray, optical and radio sources of currently uncertain origin. EM messengers that emanate from stars and dust (i.e. detectable as neither transient nor variable) in the gravitationally lensed host galaxies are also of central importance to locating transient/variable sources within them.

Interest in multi-messenger gravitational lensing has been fuelled by the breakthrough direct detections of GWs [1–4], the first multi-messenger discovery of a CBC [5–7] and the first discoveries of gravitationally lensed supernovae [8–11]. These discoveries have helped to unlock a broad range of science that spans fundamental physics, cosmology, high-energy astrophysics, nuclear physics, the chemical enrichment of the Universe and galaxy evolution. Multi-messenger gravitational lensing is well-placed to significantly expand and accelerate scientific progress in these topics (§5, and references therein).

In particular, a robust detection of a gravitationally lensed CBC via EM and GW messengers would enable novel tests of GR, providing the broadest-band large-scale laboratory for such experiments to date. GRB170817A/GW170817/AT2017gfo provided rich evidence of how multi-messenger approaches enhance discovery science [5,12]. Similarly, EM messengers associated with gravitationally lensed CBCs can make game-changing contributions by localizing the gravitationally lensed merger. Identification of the EM counterpart to a candidate gravitationally lensed GW will achieve sub-arcsecond localization in the host galaxy [13,14]. EM information about host galaxies of gravitationally lensed binary black hole (BBH) mergers can also place powerful constraints on these host galaxies and potentially achieve a similar level of precision [15–18]. The combination of sub-arcsecond angular resolution from EM messengers with the millisecond temporal resolution of the GW detectors is the key to unlocking novel science. Moreover, this exciting new lensing regime that combines superb angular and temporal resolution is also available by combining the timing precision of radio, gamma-ray and/or neutrino detections with optical/IR detection (§§2, 5c(ii) and 5c(iv)).

When considering the direct detection of multiple messengers from transient and variable sources, multi-messenger gravitational lensing is multi-messenger astronomy enhanced by multiple magnified lines of sight to sources at redshifts beyond those typically accessible without lensing. Multi-messenger astronomy itself began in the late 1980s when neutrinos were detected from a core-collapse supernova (SN1987A) in the Large Magellanic Cloud [19–21] and from the Sun [22,23]. Three decades later in 2017, the merger of a binary neutron star (BNS) and its aftermath at a distance of D=40Mpc were detected via many messengers, spanning gamma-rays to radio waves in the EM spectrum and GWs [24], and coincident neutrino and gamma-ray flares were detected from an active galactic nucleus (AGN), the Blazar TXS0506+056, at a redshift of z=0.3365 [25]. That these latter multi-messenger discoveries were sources at cosmological distances is central to demonstrating the feasibility of multi-messenger gravitational lensing discoveries (§4b), echoing the historical development of gravitational lensing.

AGN, in the form of quasars, were central to enabling the step from early work on gravitational lensing [26–30] to modern discoveries. The intrinsic brightness of quasars renders them detectable out to high redshift (z≳1) without requiring any gravitational magnification. This was key to the first discovery of a gravitationally lensed source at cosmological distances in 1979 when the quasar pair 0957+561 was confirmed as a single quasar at z=1.405 that is gravitationally lensed into two detectable images by a massive foreground galaxy at z=0.39 [31,32]. Gravitationally lensed quasars received significant impetus from the Sloan Digital Sky Survey (SDSS) in the first decade of the twenty-first century [33]. Thanks to long-term monitoring of these intrinsically variable sources ([34] for example), they now provide state-of-the-art time-delay cosmography measurements of $H_{0}$ ([35] and references therein).

GRBs are more luminous than quasars, and therefore also prime candidates for gravitationally lensed discoveries. Early discussions of gravitationally lensed GRBs were contemporaneous with establishing the extragalactic nature of most GRBs in the 1980s when Paczynski considered the gravitational lensing interpretation of three similar bursts from the source B1900+14 [36,37]. Prospects for testing the lensing interpretation of candidate-lensed GRBs improved around a decade later, following the discovery of afterglow emission from GRBs that spans X-ray to radio wavelengths [38–40] and the joint association of some supernovae (detected at optical wavelengths) and GRBs with the core collapse of massive stars [41,42]. These breakthroughs enabled the localization of GRBs to their host galaxies and thus also to the angular scale of gravitational lensing. In the modern era, *Fermi’s* Gamma-ray Burst Monitor (GBM) alone has detected greater than 3000 GRBs to date, with typical sky localization uncertainties of up to ≃$10^{3}\;degree^{2}$, of which ≃20% have arcsecond localizations via detection of an afterglow, mostly because of their co-discovery with *Swift* [43,44]. In parallel, several studies have searched for and discussed candidate gravitationally lensed GRBs, with no confirmed discoveries to date ([45–49] for example).

The first discoveries of gravitationally lensed supernovae in the mid-2010s [8–10] propelled gravitational lensing into a new regime of lensed transients—i.e. objects that subsequently fade completely and thus, unlike lensed quasars, allow detailed studies of their host galaxies. These and subsequent discoveries [11,50–52] are more highly magnified than the typical lensed quasars, because supernovae are intrinsically fainter than quasars, and therefore, at comparable detector sensitivity, they require higher magnification to be detected at cosmological distances. Importantly, in the context of multi-messenger gravitational lensing, these lensed supernovae confirmed that the discovery of gravitationally lensed optical transients is feasible. Moreover, the gain in survey sensitivity from Rubin/LSST will drive significant growth in the number of discoveries in the coming decade [53–56] and motivate the optimization of discovery methods for gravitationally lensed optical transients relevant to multi-messenger gravitational lensing [57–63].

Following the first direct detection of GWs [1] by the ground-based network that now comprises the two LIGO detectors [64], the Virgo detector [65] and the KAGRA detector [66], signatures of gravitational lensing were discussed and searched for both by the LIGO–Virgo–KAGRA (LVK) collaborations and groups external to the LVK [67–80]. These drove the development of several analysis methodologies ([69,74,81–92] for example), in addition to forecasts of the rate of detection of lensed CBCs [13,93–100]. The first direct detection of GWs was swiftly followed by the first multi-messenger detection of a CBC ([5,6,12,24,101–104] and references therein). In the intervening years, several scientific applications of multi-messenger gravitational lensing discoveries were proposed, including tests of GR [105,106], the speeds of light and GWs [107–109] and measurements of the expansion of the Universe ([110–112] for exxample).

To facilitate the multi-messenger discovery of gravitationally lensed CBCs, significant attention has focused on EM follow-up observations of GW sources with masses that are consistent with the lensing hypothesis [13,57,58,99,113–119]. The aim of these studies, given the proven association of GRBs and kilonovae with BNS mergers [6], is to localize candidate-lensed GW sources to sub-arcsecond accuracy within their respective gravitationally lensed host galaxies, via detection of a lensed EM counterpart. There are also intriguing claims that some BBH mergers might have EM counterparts in the form of AGN flares caused by a merger occurring in an AGN accretion disk [120–124]. If AGN flares are confirmed as EM counterparts to BBH mergers, this may lead to the gravitational lensing of stellar remnant CBCs by the AGN or galaxies/groups/clusters that intervene along the line of sight [119,125]. Identification of the host galaxies of BBH mergers without EM counterparts has also been investigated, via comparison of the properties of known gravitational lenses derived from EM surveys with candidate gravitationally lensed BBH mergers [15–18].

FRBs, first discovered in 2007 [126], are located at cosmological distances and are of intriguing unknown origin [127–129]. The rapidly growing numbers of detections, already in the hundreds, and the timing and sky localization accuracy of the detections identify them as exciting and relevant for multi-messenger gravitational lensing discoveries. Indeed, numerous works have explored the potential for gravitationally lensed FRBs to probe the nature of DM, to test fundamental physics including GR and to elucidate the putative connection between FRBs and sources of GWs ([130–138] for example).

Multi-messenger gravitational lensing discovery and science span a diverse community and many disciplines. A significant fraction of the community came together for the first time in Manchester on 11−12 March 2024 at a Theo Murphy Discussion Meeting hosted by The Royal Society. This meeting focused mainly on opportunities in the upcoming decade with facilities that survey a large fraction of the celestial sphere, including *Fermi*, Rubin/LSST and LVK. This article captures the consensus that emerged in Manchester and aims to share it with the wider community. We give an overview of the relevant multi-messenger signals (§2), outline the essentials of gravitational lensing theory and phenomenology (§3), describe the multi-messenger gravitational lensing discovery channels, discovery rates and key challenges (§4) and present the main multi-messenger gravitational lensing science cases (§5).

## 2. Multi-messenger signals and instruments

The messengers span at least 30 orders of magnitude in energy (table 1), from high-energy neutrinos ($E_{\nu }≳10^{15}\mathrm{eV}$) through to low-frequency GWs ($E_{\mathrm{GW}}=hf≲10^{-15}\mathrm{eV}$, where f is the GW frequency). This vast range of energy is mirrored by differences in the technology required to detect the messengers, the relative sensitivities of instruments across the energy scale and how the messengers complement each other in the context of gravitational lensing. We refer the interested reader to review articles in this volume and elsewhere, for further details of the physics of each messenger, how they are detected and the science questions that each messenger is well-suited to probing [12,63,163–166].

**Table 1.** Summary of messengers, multi-messenger transient and variable sources, survey instruments (angular grasp of ≳2πsr) and prospects for gravitationally lensed discoveries in the coming decade.

Messengers and sources^a^	Detections to date^b^			Expectations in next decade^c^			References
	$N_{\mathrm{tot}}$	⟨z⟩	$N_{\mathrm{lensed}}^{\det}$	Detector/facility	$z_{H}$	$N_{\mathrm{lensed}}^{\mathrm{pred}}$	
Flux-like messengers							
Neutrinos	*[Timing accuracy^d^, $\sigma_{t}≃10^{-9}s$ ; Sky localization uncertainty^e^, $\Delta \Omega ≲5\;degree^{2}$ ]*						
Blazar	1	0.34	0	IceCube-Gen2	1	1	[25,139]
Extended emission GRB	0	0.03	0	IceCube-Gen2	0.07	<1	[140]
Millisec. magnetar	0	0.002	0	IceCube-Gen2	0.02	<1	[141]
Core-collapse SN	1	$10^{-4}$	0	Hyper-Kamiokande	0.001	<1	[142,143]
BBinary NS merger	0	…	0	Hyper-Kamiokande	$10^{-4}$	<1	[144,145]
Gamma- and X-rays	*[ $\sigma_{t}≃10^{-3}s$ ; $\Delta \Omega ≃10^{-2}-10^{4}degree^{2}$ ]*						
Long GRB	$10^{4}$	3	0	*StarBurst, SVOM*	3	10	[146]
Short GRB	$10^{3}$	1	0	*StarBurst, SVOM*	1.5	1	[147]
Relativistic TDE	< 10	1	0	*Einstein Probe, SVOM*	6	< 1	[148]
Fast X-ray transients	100	3	0	*Einstein Probe, SVOM*	3	< 1	[149,150]
Optical and near-IR^f^	*[ $\sigma_{t}≃10^{4}-10^{6}s$ ; $\Delta \Omega ≃10^{-8}degree^{2}$ ]*						
Super-luminous SN	300	0.4	0	LSST WFD	1.7	> 10	
Type Ia SN	> $10^{4}$	0.3	2	LSST WFD	0.8	> 100	[53–55]
TDE	100	0.3	0	LSST WFD	0.7	10	[151,152]
Core-collapse SN	> $10^{3}$	0.2	0	LSST WFD	0.5	> 100	[53,54]
GRB afterglow	> $10^{3}$	0.1	0	LSST WFD/ToO^g^	0.3/0.3	10/10	[119]
Kilonovae	10	0.1	0	LSST WFD/ToO	0.2/0.7	1/1	[119,153,154]
amplitude-like messengers							
Radio waves	*[ $\sigma_{t}≃10^{-3}s$ ; $\Delta \Omega ≃10^{-8}degree^{2}$ ]*						
FRB	$10^{3}$	1	0	CHIME/FRB, CHORD	3	10	[155]
GRB afterglow	> 400	1	0	SKA-Mid	5	10	[156,157]
Gravitational waves	*[ $\sigma_{t}≃10^{-3}s$ ; $\Delta \Omega ≃10-10^{4}degree^{2}$ ]*						
Binary BH merger	>90	0.4	0	LVK A ${,}^{+}$/A ${,}^{♯}$ (XG)	2/5(40)	>1/5(50)	[158–161]
NS-BH merger	3	0.1	0	LVK A ${,}^{+}$/A ${,}^{♯}$ (XG)	0.3/0.6(20)		[158–162]
Binary NS merger	2	0.04	0	LVK A ${,}^{+}$/A ${,}^{♯}$ (XG)	0.2/0.4(8)	<1/1(50)	[158–161]
Core-collapse SN	0	$10^{-5}$	0	LVK A ${,}^{+}$/A ${,}^{♯}$ (XG)	$\left(10^{-4}\right)$		[161]

^a^ Messengers (underlined) are listed in order of decreasing energy scale, and under each messenger, the sources are listed in order of decreasing intrinsic brightness.

^b^ Summary of the detections to date by wide-angle survey facilities with sustained operations that span years and at least half of the celestial sphere. *N*_tot_ is the total number of sources detected to date, ⟨z⟩ is the typical redshift of the detected sources (approximate peak of the redshift distribution of a SNR ratio limited sample; not a formally computed mean) and *N*_lensed_ is the number of confirmed gravitationally lensed sources detected to date by these wide-angle surveys.

^c^ Wide-angle surveys and detectors that have come online recently, or will do so in the next decade, with their sensitivity summarized by the expected redshift horizon out to which they can detect sources without assistance from gravitational magnification (zH), and order of magnitude expected number of lensed detections in the next 10 years. For GWs, we quote the expected number of events per year with three different detector sensitivities: 2 expected upgrades of LVK detectors (A^+^/A^♯^) and 1 for next-generation (XG) ground-based detectors such as Einstein Telescope and Cosmic Explorer.

^d^ The accuracy with which the arrival time of the transient signals can be measured, σ_t_. This is set by the properties of the detectors for all messengers except optical/near-IR messengers, which are limited by the shape of their light curve. For example, faster transients (e.g. kilonovae) have σ_t_ ≃ 10^4^ s, the slowest transients (e.g. super-luminous supernovae) have σ_t_ ≃ 10^6^ s, and GRB afterglow light curves do not constrain arrival time.

^e^ The uncertainty in the sky localization of the messenger. For surveys that use reflecting optics, this is given as the solid angle subtended by a circle of diameter comparable with the full width at half maximum of point sources. For all other surveys, it is given as the solid angle of the typical 90% confidence interval on the sky.

^f^ The following peak absolute magnitudes have been adopted: SLSN = −21.5, Type Ia Supernova (SNIa) = −19.4; TDE = −19.3; Core-Collapse SN (CCSN) = −18; GRB afterglow = −17; AT2017gfo-like kilonova = −15.7; Conservative KN = −14.5; NS-BH KN = −13.0. The assumed sensitivity of ongoing optical surveys is an apparent magnitude of *m* = 20, i.e. approximately matching the depth of PanSTARRS, ATLAS, ZTF, GOTO, LS4 and BlackGEM.

^g^ The assumed sensitivity of Rubin/LSST ToO observations is *m* = 24 for lensed GRB afterglow counterparts to candidate-lensed GRBs and *m* = 27 for lensed kilonova counterparts to candidate-lensed BNS mergers, respectively [119].

A key distinction between different messengers is whether they are detected via flux or amplitude (see ‘Flux-like’ and ‘Amplitude-like’ sections of table 1) and among those detected via flux whether or not the individual particles/photons energies are measured. This is important in the context of gravitational lensing because gravitational magnification (μ) describes the transformation of solid angle (§3a), therefore flux scales with μ and wave amplitude scales with $\sqrt{\mu }$. Neutrino and gamma-ray instruments (e.g. IceCube, Kamiokande, *Fermi* and *Swift*) count and measure the energy of individual particles and photons, while most optical and IR instruments (e.g. PanSTARRS, ZTF, Rubin/LSST) count photons without measuring their individual energies. These messengers are therefore detected primarily via the flux of energy that arrives at the respective instruments. Lower energy messengers are detected via their wave amplitude. Radio instruments measure radio wave amplitude via the time-varying voltages that they detect (e.g. CHIME/FRB). GW instruments detect the amplitude of GWs that arrive at Earth via the strain signal that is measured with interferometers. While detection does not rely on whether messengers are polarized, all of them can, in principle, be polarized, and this can lead to important science applications (e.g. §5a(iv)).

The timing accuracy and sky localization uncertainty of the instruments differ dramatically between the messengers (table 1). Broadly speaking, superb timing accuracy ($\sigma_{t}<1\;s$) is associated with poor sky localization uncertainties ($\Delta \Omega >10\;degree^{2}$) and vice versa. This is important because combining multiple gravitationally lensed messengers that have complementary strengths in timing and sky localization has great potential to unlock discoveries (§4) and novel science (§5). Discovery and science are enhanced by the direct detection of different messengers from an EM-bright gravitationally lensed transient/variable source (§4c(i)). A complementary approach uses optical information about galaxies located behind known gravitational lenses to search for lenses responsible for pairs of EM-dark GW detections that are lensed images of the same source (§4c(ii)).

To be more specific, the superb timing accuracy of gamma-ray, radio and GW detection complements the superb angular resolution (sky localization uncertainties) of optical/IR transient surveys through which lensed optical counterparts can be identified. The latter can be further significantly enhanced by the astrometric precision that can be achieved with follow-up observations using the *Hubble Space Telescope* and *James Webb Space Telescope*. Measurements of the arrival times of optical/IR signals stand out in table 1 as the least accurate among the messengers. The accuracy of optical measurements is currently set by the measurement uncertainties on when the respective peaks—typically of order days. Interferometric radio detection also stands out in table 1, as the only messenger for which accurate sky localization *and* arrival time difference measurements are feasible. These features point to exciting prospects for scientific exploitation of gravitationally lensed FRBs, especially when combined with other messengers (§5c(iv)) [163].

The redshift horizons, $z_{H}$, listed in table 1, indicate the maximum redshifts at which sources are detectable in the coming decade via each messenger without assistance from gravitational magnification. The numbers of gravitationally lensed sources that are forecast to be detected in the coming decade roughly scale with $z_{H}$ because, for reasonable assumptions on the comoving rate density of sources, a larger value of $z_{H}$ indicates a larger comoving volume within which detectable sources may be located. This has important consequences for the focus and balance of this article and is explained in detail in §4. In summary, we focus on messengers for which $z_{H}≳0.1$, as these offer the strongest potential for discovery of lensed sources in the coming decade.

## 3. Gravitational lensing theory and phenomenology

We give an overview for the non-expert of gravitational lensing theory and phenomenology in the context of multi-messenger astronomy and refer readers to other works ([167,168] for example), and others cited below for further theoretical details.

### (a) Arrival time, deflection and magnification

The travel time, t, from a source at a redshift of $z_{S}$ along a null geodesic through a gravitational field at a redshift of $z_{L}$ depends on distances and the Fermat potential of the lens, τ:


(3.1) $$\begin{array}{lcr} & c\;t=\left(1+z_{L}\right)\frac{D_{L}D_{S}}{D_{\mathrm{LS}}}\;\;\tau (\theta ,\beta ), & \end{array}$$

where $D_{L}$, $D_{\mathrm{LS}}$ and $D_{S}$ are the angular diameter distances from the observer to the lens, lens to source and observer to source, respectively, β is the true position of the source on the celestial sphere and θ is the position of the gravitationally lensed image of that source.

The Fermat potential comprises the geometrical path length difference between the unperturbed observer–source path and the actual path (first term on the right-hand side of equation (3.2)), and a relativistic term [169] that is described by the deflection potential of the lens, ψ (second term):


(3.2) $$\begin{array}{lcr} & \tau (\theta ,\beta )=\frac{(\theta -\beta {)}^{2}}{2}-\psi (\theta ). & \end{array}$$

The deflection potential satisfies the two-dimensional Poisson equation, $\nabla^{2}\psi =2\kappa$, where $\kappa \equiv \Sigma /\Sigma_{\mathrm{crit}}$ is the dimensionless projected matter density of the lens and $\Sigma_{\mathrm{crit}}$ is the critical density given by


(3.3) $$\begin{array}{lcr} & \Sigma_{\mathrm{crit}}=\frac{c^{2}}{4\pi G}\;\frac{D_{S}}{D_{L}D_{\mathrm{LS}}}. & \end{array}$$

In practice, the arrival time *difference* between two gravitationally lensed images ($\Delta t_{\mathrm{AB}}=t_{A}-t_{B}$) and the positions of the images ($\theta_{A},\theta_{B}$) are measurable if the measurement uncertainties are sufficiently small, while the difference between the unperturbed travel time and either $t_{A}$ or $t_{B}$, and also β, are intrinsically not measurable. The arrival time difference is therefore conventionally written as


(3.4) $$\begin{array}{lcr} & \Delta t_{\mathrm{AB}}=\frac{D_{\Delta t}}{c}\left[\tau (\theta_{A},\beta )-\tau (\theta_{B},\beta )\right], & \end{array}$$

where $D_{\Delta t}$ is the so-called time-delay distance that is defined as


(3.5) $$\begin{array}{lcr} & D_{\Delta t}\equiv (1+z_{L})\frac{D_{L}D_{S}}{D_{\mathrm{LS}}}. & \end{array}$$

By construction, the time-delay distance is inversely proportional to $H_{0}$ and is central to time-delay cosmography (§5b(i)).

Applying Fermat’s Principle to equation (3.2) (i.e. requiring ∇τ=0) yields the locations of the image(s) of gravitationally lensed sources, i.e. the lens equation:


(3.6) $$\begin{array}{lcr} & \theta =\beta +\nabla \psi (\theta )=\beta +\alpha (\theta ), & \end{array}$$

where α=∇ψ is the deflection angle. Strong lensing—the formation of multiple images—corresponds to multiple solutions, $\theta_{k}$, of equation (3.6) for a given source position, β.

Most gravitational lenses are approximately axially symmetric with κ decreasing as a function of angular offset from the lens centre, θ=|θ|, and produce multiple images of distant sources at lens-centric angles that satisfy ⟨κ(<θ)⟩=1 [170]. These images form at or close to the so-called Einstein radius, $\theta_{E}$, which is defined as follows for an axially symmetric lens:


(3.7) $$\begin{array}{lcr} & \theta_{E}={\left(\frac{4GM}{c^{2}}\;\frac{D_{\mathrm{LS}}}{D_{L}D_{S}}\right)}^{1/2}\;, & \end{array}$$

where $M=M(<\theta_{E})$, i.e. the projected mass interior to the Einstein radius.

The flux that arrives at Earth from a gravitationally lensed source differs from the flux that would arrive in the absence of gravitational lensing by a factor |μ|, where μ is the lens magnification:


(3.8) $$\begin{array}{lcr} & \mu ={\left[(1-\kappa {)}^{2}-\gamma^{2}\right]}^{-1}. & \end{array}$$

where κ and γ are the convergence and shear, respectively—i.e. the isotropic and anisotropic contributions to the magnification. They are related to the second-order partial derivatives of the deflection field: $\kappa =(\psi_{,11}+\psi_{,22})/2=\nabla \psi^{2}/2$ (as above), $\gamma_{1}=(\psi_{,11}-\psi_{,22})/2$ and $\gamma_{2}=\psi_{,12}=\psi_{,21}$. The subscripts on ψ denote partial differentiation with respect to the two components of θ.

The amplitude of a gravitationally lensed wave-like signal therefore differs from the intrinsic amplitude of the signal by a factor $\sqrt{|\mu |}$. As a consequence, both flux- and wave-like signals that are gravitationally magnified are apparently brighter/louder than the underlying source if |μ|>1.

### (b) Critical curves, caustics and image parity

The multiple images that are created by strong gravitational lenses form adjacent to so-called ‘critical curves’ in the image plane that is accessible to our detectors. These curves are closed and are analogous to the perfect Einstein rings of radius $\theta_{E}$ associated with a hypothetical axially symmetric lens. A critical curve is the boundary between regions of positive and negative gravitational magnification, where the sign indicates the parity of the gravitational image formed in that region. In this context, parity refers to the handedness of the image, as seen in the mirror symmetry of a pair of optical images, or phase of the GW signal associated with images that form on either side of a critical curve.

Critical curves map to caustics in the source plane that demarcate the regions of different image multiplicity, such that if a source is exterior to all caustics, it produces one image of positive parity and it produces additional pairs of images (of no net parity per pair) for every caustic within which it is located. Images formed at minima and maxima of the arrival time surface have positive parity and are called Type I and III images, respectively, while images formed at saddle points have negative parity and are called Type II images.

### (c) Achromaticity

Gravitational lensing is achromatic in the geometrical optics limit (§3d), i.e. the spectrum of a source is unaltered by gravitational lensing. At optical wavelengths, this enables multiple images of gravitationally lensed galaxies and explosive transients to be identified via similarity in their broad-band colours and/or spectra, in addition to mirror symmetries due to parity conservation (§3b). Achromaticity is also key to identifying strongly gravitationally lensed GWs. The multiple images of a GW source are broadly identical in frequency evolution, which enables multiple images of a GW source to be identified in the time domain, and the arrival time difference between these images to be measured. Each image does, however, experience its own frequency-independent phase shift, which can alter waveform morphology in some cases, depending on the image type [171,172]. This caveat can also be exploited to identify Type II images in cases where higher order modes are present in the GW signal [173–175].

Achromaticity may not hold in several scenarios that are relevant to multi-messenger gravitational lensing. First, the spectral similarity of multiple gravitational images of a source assumes that emission from the source is isotropic on angular scales probed by the multiple sight lines to the source afforded by lensing. For compact sources such as supernovae or CBCs, achromaticity therefore assumes isotropy of emission on the scale of the Einstein radius of the gravitational lens, i.e. θ≲1arcmin. This is discussed in the context of the least isotropic messenger considered in this article—gravitationally lensed GRBs—in §5c(ii). Second, gravitational magnification can be frequency-dependent in scenarios where the geometrical optics limit breaks down, for example, GWs that are lensed by compact lenses such as stars and compact objects [176–180]. In such cases, a ‘wave optics’ treatment is necessary (§3d). Note also that an additional frequency-dependent modulation can arise in strongly lensed images when those encounter smaller objects present in the lens [84,176,177,179,180]. Third, micro-lensing may also affect the light curves of lensed optical counterparts to lensed GW sources, causing systematic differences between the photometric evolution of different lensed images of the same source. This has, for example, been investigated in the context of measuring arrival time differences from gravitationally lensed SNe ([181] and references therein).

### (d) Physical scales of lensing, and geometric, Eikonal and wave optics

Gravitational lensing presents several phenomenological differences that depend on the physical scale of the lens and the messenger being considered. First, lensing by massive extended objects such as galaxies or galaxy clusters—in which the source is inside the caustic of the lens—is referred to as strong lensing. Such objects are typically hosted by DM halos that span at least $M_{200}≃10^{12}-10^{15.5}\;M_{⊙}$ [182,183], where $M_{200}$ is the mass in the spherical region within which the mean density is 200× the critical density of the Universe. This produces multiple images that are potentially both spatially and temporally resolvable. As is common in the literature, we use the term ‘images’ to denote the repeated signals for gravitationally lensed sources regardless of the nature of the messenger.

Moving down in physical scale while keeping the source within the caustic of the lens, the angular–temporal separation between the images shrinks, eventually leading to images that overlap and then images that are no longer individually resolvable [184]. This is the domain of millilensing and microlensing, typified by angular separations of milli- and micro-arcseconds, respectively. Millilensing/microlensing and strong lensing are not mutually exclusive, because small-scale lenses can be present in large extended lenses, and thus perturb the strong lensing signal ([185] and references therein). Moreover, the magnifying effect of a strong lens can boost the detectability of millilensing/microlensing [178].

Finally, weak lensing refers to the imprint of the gravitational field of a lens on the signals from distant sources that are located well outside the caustics discussed in §3b [186,187]. Weak lensing, therefore, does not produce multiple images of distant sources. At optical wavelengths, weak lensing causes subtle distortions in the measured shapes of distant galaxies. Measurement and analysis of weak lensing signals require careful statistical analysis of a large number of sources due to the subtle effects being unmeasurable for individual sources. While we do not focus on weak lensing here, it is also a source of bias in standard siren cosmology ([188] for example). Efforts to control this bias can benefit from the enhanced knowledge of the host galaxies of GW sources that can be obtained from multi-messenger gravitational lensing detections (§5c(v)).

**Figure 1.**
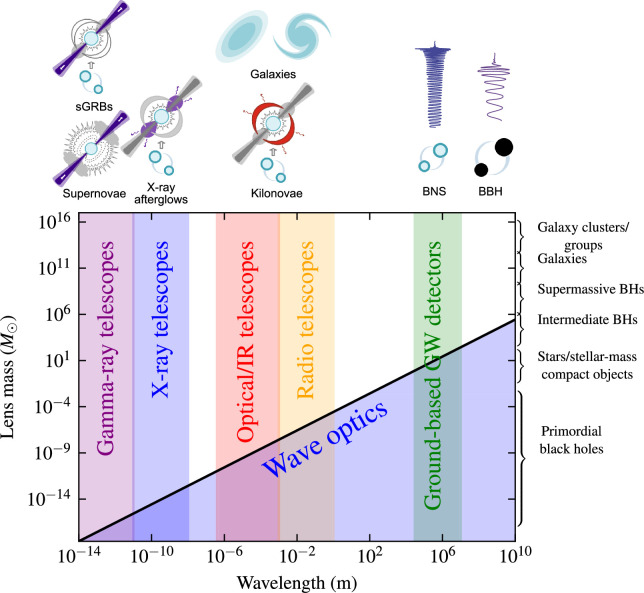
Illustration of the mass scales at which wave optics effects become relevant for gravitationally lensed signals. The geometric optics regime is valid when the wavelength of the radiation is much smaller than the scale of the lensing potential. The wave optics regime is valid when the wavelength is comparable to the scale of the lensing potential. Because the wavelength of GWs detected by the current ground-based detectors is typically much larger than the wavelength of most light sources, wave optics effects can become relevant for lenses below $≲100\;M_{⊙}$. Since other GW detectors like *LISA* will be sensitive to even longer wavelengths, wave optics effects will be even more important. The precise mass scale also depends on the lensing configuration, such as the distance from the caustic, where wave optics effects can become more prominent close to a caustic when the magnification is large.

Turning to the physical treatments, three regimes are relevant: geometric, wave and Eikonal optics. Geometrical optics is relevant if the wavelength of the messenger is much smaller than the physical scale of the lensing potential. It applies to both EM and GW messengers (figure 1) and is described in §3a−c. Wave optics is relevant if the wavelength of the messenger is larger than or comparable with the physical scale of the lensing potential. This applies to GWs that are detectable by ground-based detectors, specifically for which the GW wavelength, $\lambda_{\mathrm{GW}}$, is comparable with the Schwarzschild radius, $R_{S}=2GM/c^{2}$, of the lens. In such cases, effects such as diffraction must be included in the treatment [189–192], and the total magnification of the GW waveforms must be fully computed as [190]


(3.9) $$\begin{array}{lcr} & F(f)=\frac{1+z_{L}}{c}\frac{D_{L}D_{S}}{D_{\mathrm{LS}}}\frac{f}{i}\int d^{2}\theta \;\exp[2\pi if\Delta t(\theta ,\beta )]\;. & \end{array}$$

For the current GW detector network, wave-optics effects are relevant for compact lenses with masses $≲100\;M_{⊙}$. Wave-optics effects will be relevant at much higher lens masses in the future when space-based detectors such as *LISA* [193] probe longer wavelengths, for example, from binary supermassive black holes [191,194–197].

Eikonal optics refers to the regime in which the arrival time differences between the multiply-imaged GW signals are less than the duration of the signal. In this regime, interference between the multiple signals must also be considered. This can occur either due directly to the mass of the object [198] or in the case of highly magnified images ([13,192,199–202] for example). In the latter case, for a representative galaxy-scale lens with $\theta_{E}≃1\;\mathrm{arcsec}$, this corresponds to gravitational magnifications of μ≳50 for a quad image configuration. For fold image pairs produced by a representative galaxy cluster lens ($\theta_{E}≃5\;\mathrm{arcsec}$), this corresponds to μ≳200 (§3g; [13]).

### (e) Mass sheet degeneracy

Robust physical interpretation of gravitationally lensed signals requires the so-called ‘mass sheet degeneracy’ to be broken [203,204]. This degeneracy affects the inference of the properties of the lens mass distribution, the size and luminosity of sources and cosmological parameters, including $H_{0}$. Put simply, if a given projected mass distribution κ(θ) gives an adequate fit to the measured image positions, flux ratios and any measured arrival time differences, then so too do the rescaled mass distributions


(3.10) $$\begin{array}{lcr} & \kappa_{\lambda }=(1-\lambda )+\lambda \;\kappa (\theta ), & \end{array}$$

where λ is an arbitrary scalar.

The three gravitational lensing phenomena of arrival time, deflection and magnification are affected differently by the mass sheet degeneracy. The arrival time difference between two transient or time-varying signals is altered by $\Delta t_{\lambda }=\Delta t\;\lambda$ and, as implied above, the image positions are unaltered, but the source positions (and by association also the deflection angles) are altered by $\beta_{\lambda }=\beta \;\lambda^{-1}$, while the magnification is altered by $\mu_{\lambda }=\mu \;\lambda^{-2}$, where subscript λ denotes quantities related to the rescaled density field. The mass sheet degeneracy can be broken if independent information is available about the mass of the lens, e.g. from stellar dynamics, the size or luminosity of the source, the characteristic interference patterns of GW waveforms or for multiple source planes behind the same lens ([55,200,205–209] for example).

### (f) Galaxy-scale strong lenses

Galaxy-scale strong lenses discovered to date in optical imaging surveys are typically early-type galaxies with Einstein radii of $\theta_{E}≃1\;\mathrm{arcsec}$ ([182] and references therein). These discoveries are based on recognizing gravitationally lensed sources as multiply-imaged quasars and/or gravitational arcs in the absence of time-domain information. Time domain surveys offer complementary selection methods that can exploit the arrival time difference between images of lensed explosive transients [53,55,101]. For example, recent discoveries of strongly lensed supernovae probe a population of lenses with sub-arcsec Einstein radii [10,63].

Galaxy-scale lenses typically form two or four detectable images—so-called double and quad lenses, respectively. A third or fifth image, respectively, is strongly demagnified and located close to the centre of the lens. The basic properties of galaxy-scale lenses are well described by an isothermal density profile


(3.11) $$\begin{array}{lcr} & \kappa (x)=\frac{1}{2x}, & \end{array}$$

where $x\equiv \theta /\theta_{E}$ [210]. While galaxy-scale lenses are approximately axially symmetric, formally the typical model of a galaxy-scale lens is an ellipsoidal power law [182].

Double images arise from sources at $y\equiv \beta /\theta_{E}<1$ from the centre of a galaxy-scale lens. The arrival time difference between, angular separation of and total magnification of the image pair are given by


$$\begin{array}{lrlr} & \frac{\Delta t_{\text{double }}}{92\text{ days }} & ={\left[\frac{\theta_{E}}{1^{\prime \prime }}\right]}^{2}\left[\frac{y}{0.5}\right]\left[\frac{D_{\Delta t}}{3.3\mathrm{Gpc}}\right] & \\ & \Delta x_{\text{double }} & =\left|x_{+}-x_{-}\right|=2, & \left(\text{3.12}\right)\\ & \mu_{\text{double }} & =\left|\mu_{+}\right|+\left|\mu_{-}\right|=\left(1+\frac{1}{y}\right)+\left(\frac{1}{y}-1\right)=\frac{2}{y}, & \end{array}$$

where $x_{\pm }$ and $\mu_{\pm }$ denote the positions and magnification of each image, respectively. Formally, the threshold for multiple image formation is $\mu_{\mathrm{double}}=2$, for a source at y=1; however, in this case, one image is not detectable because $\mu_{-}=0$. Sources that are more closely aligned with the centre of the lens produce more highly magnified double images with shorter arrival time differences. For example, at y<0.5, both images are brighter than the source, with $\mu_{+}>\mu_{-}>1$.

Quad images arise because galaxy-scale strong lenses are typically elliptical, creating a caustic that can produce an additional image pair if the source lies inside it. This caustic is typically located at y<1, and has a characteristic astroid shape that comprises four cusps connected by smooth curves that are called fold caustics. Following the formalism introduced by [168], the arrival time difference between, angular separation of and magnification of each image of a fold image pair can be expressed as


Δtfold0.25 days =[Υt1][Δy00.01]1.5[θE1′′]2[DΔt3.3Gpc](3.13)Δxfold0.4=[Υx1][Δy00.01]0.5μfold 10=[Υμ1][Δy00.01]−0.5,

where $\Delta y_{0}=\Delta \beta_{0}/\theta_{E}$ is the length of the shortest arc that connects the source position with the fold caustic, the density profile of the lens local to the image plane position that corresponds to $\Delta y_{0}=0$ is given by $\kappa =\kappa_{0}x^{\eta_{0}}$, and $\Upsilon_{t}$, $\Upsilon_{x}$ and $\Upsilon_{\mu }$ describe the density and structure of the lens at the mid-point of the shortest arc that connects the image pair:


(3.14) $$\begin{array}{lcr} & \Upsilon_{t}=\Upsilon_{x}=\Upsilon_{\mu }\;|\eta_{0}{|}^{0.5}=[|\eta_{0}|\;(2+\eta_{0}){]}^{-0.5}, & \end{array}$$

where $-1<\eta_{0}<0$, and equation (3.13) relies on the relation $\kappa_{0}=1+\eta_{0}/2$ for approximately axially symmetric lenses to eliminate $\kappa_{0}$. For an isothermal galaxy-scale lens $\eta_{0}=-1$ and $\kappa_{0}=0.5$ equation (3.11), yielding $\Upsilon_{t}=\Upsilon_{x}=\Upsilon_{\mu }=1$.

Quad images eare a higher magnification regime than double images because the source needs to be more closely aligned with the high-magnification central region of the lens to access the fold caustic. Quads are therefore also associated with shorter arrival time differences than doubles, because arrival time difference scales inversely with magnification, with stronger scaling for folds than for doubles: $\Delta t_{\mathrm{fold}}\propto \mu_{\mathrm{fold}}^{-3}$, $\Delta t_{\mathrm{double}}\propto \mu_{\mathrm{double}}^{-1}$.

### (g) Group/cluster-scale strong lenses

Galaxy groups and clusters have typical Einstein radii in the range $\theta_{E}≃3-60\;\mathrm{arcsec}$, i.e. larger than individual early-type galaxy-scale lenses, due to the enhanced projected density in group and cluster cores that is attributable to the massive DM halo ($M_{200}≃10^{13}-10^{15.5}\;M_{⊙}$) in which they are embedded [183]. The DM contribution causes the density profiles of group- and cluster-scale lenses to be denser ($\kappa_{0}>0.5$) and flatter ($\eta_{0}>-1$) than isothermal at their Einstein radii [211,212]. This reduces the efficiency of clusters in producing multiple images in the low-magnification regime that is accessible to galaxy-scale doubles, i.e. μ≲10 [13]. A similar effect is expected for group-scale lenses; however, this has not yet been studied in detail. The cores of group- and cluster-scale lenses tend to be strongly asymmetric, and thus fold caustics tend to dominate the multiple images that they form. This is particularly true for massive galaxy clusters due to the prevalence of substructures resulting from the hierarchical nature of large-scale structure ([213–217] for example).

The arrival time difference, image separation and individual image magnifications of fold image pairs formed by group and cluster lenses are also given by equation (3.13). Given the density and structure of group- and cluster-scale lenses discussed above, $\Upsilon_{t}$, $\Upsilon_{x}$ and $\Upsilon_{\mu }$ all tend to exceed unity and thus the arrival time difference, image separation and magnification of fold image pairs are all larger for group/cluster-scale lenses than for galaxy-scale quad lenses. In summary, the phenomenology of group and cluster lenses relative to galaxy lenses can be understood broadly in terms of how their density profiles and substructures shape their fold caustics and efficiency of multiple image formation at μ≲10.

### (h) Compact lenses

Compact lenses also form images on the angular scale of their Einstein radius (equation (3.7)). The total magnification of these images, formed by an isolated compact lens, is given by


(3.15) $$\begin{array}{lcr} & \mu =\frac{y^{2}+2}{y\sqrt{y^{2}+4}}, & \end{array}$$

where y is the dimensionless impact parameter and has the same definition as in §3f.

When compact lenses are embedded in a dense environment such as stellar fields or galaxy/group/cluster-scale lenses, the lensing effects of compact lenses can be enhanced, leading to caustic networks and complex lensing patterns [176–180,218]. Compact lenses are therefore crucial in studying DM distributions, distant stars and black holes. By tracing their gravitational signatures, they offer insights into the unseen mass in the Universe, such as primordial black holes or other forms of DM.

### (i) Optical depth

The optical depth to gravitational lensing, τ, is defined as the fraction of the celestial sphere that is gravitationally lensed. It is useful to define it in the source plane, i.e. the fraction of the intrinsic celestial sphere, because this is well-suited to predicting and interpreting the number of gravitational lens discoveries. The source plane optical depth can be defined in terms of the number of gravitationally lensed sources that will be detected [168], or in terms of the number of images that will be detected [115,219]. Formally, our overview in this section considers the latter because it is arguably better aligned with the focus of this article, with each detectable gravitationally lensed image representing an opportunity to make the first multi-messenger discovery of a gravitationally lensed source.

Defined in this way, the optical depth as a function of the mass (M) and redshift of the lenses ($z_{L}$) can be written as follows:


(3.16) $$\frac{\partial^{2}\tau }{\partial M\partial z_{L}}=\frac{1}{\Omega }\frac{\partial^{2}\sigma_{tot}}{\partial V\partial M}\frac{dV}{dz_{L}},$$

where Ω=4π is the solid angle of the celestial sphere, $\sigma_{tot}$ is the sum of the cross-sections of all the gravitational lenses in the mass interval dM, in the comoving volume element dV. Using this equation requires the cross-section of gravitational lensing to be defined across the relevant mass and redshift range.

Gravitational lenses span a wide range of mass and internal structure that affect the arrival time differences, image separations and magnifications of the gravitational images that they produce (§3f−h). One approach is to define the cross-section in terms of multiple-image formation and assume that all lenses are early-type galaxies, with the number and masses of the lenses normalized to the SDSS galaxy velocity dispersion function ([54,55,69,220] for example). The main advantage of this approach is that by concentrating exclusively on isothermal lenses, it enables self-consistent predictions of arrival time differences, image separations and magnifications. The main disadvantage is that it ignores the impact of shallower than isothermal group/cluster-scale density profile slopes on the efficiency of multiple-image formation, arrival time differences, image separations and magnifications (equation (3.13)).

An alternative is to build the optical depth on cosmological *n*-body simulations. Such approaches include ray tracing through DM halos from the Millennium simulation in which analytic galaxies have been pasted [219], deriving the optical depth to gravitational magnification from cosmological hydrodynamical simulations [115] and using halo models calibrated to cosmological simulations to extend the galaxy-scale lens approach to account for their host DM halos [221]. The main advantage of these methods is that in principle they incorporate the full range of lens mass and structure, and thus address the disadvantage of the galaxy-scale methods outlined above.

**Figure 2.**
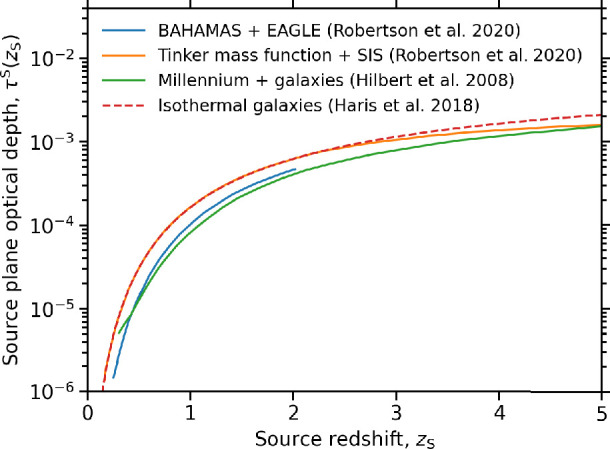
Different models of the source plane optical depth to gravitational lensing agree within a factor ≃2. This indicates that the integral of the optical depth across the mass function of lenses is converged. As discussed in the text, the distribution of the optical depth across the mass function is less well converged and is a key area for theoretical and observational progress. Figure reproduced from [13].

The approaches outlined above tend to agree within a factor of ≃2 on the integral over the mass function of the optical depth to gravitational magnification as a function of source redshift (figure 2). This encourages confidence in the following expression adapted from [69]:


(3.17) $$\frac{d\tau \left(z_{S}\right)}{d\mu }={\left(\frac{D_{S}\left(1+z_{S}\right)}{62.2Gpc}\frac{2}{\mu }\right)}^{3},$$

where the first term in parenthesis on the right-hand side describes the optical depth to magnification μ=2, and the second term scales that to higher magnifications. However, the approaches outlined above tend to disagree on how the optical depth is distributed with respect to mass and therefore with respect to lens structure. This is either by construction in the case of the galaxy-only models or likely due to differences in the implementation of baryons in the simulation-based methods, as discussed, for example, by [115].

## 4. Discovery channels for multi-messenger gravitational lensing

The path to the first multi-messenger gravitational lensing discoveries depends on synergies between the messengers that go beyond the detectability of gravitationally lensed transient sources via different messengers (§2). In this section, we describe how these synergies shape several complementary channels through which the first discoveries will be made.

We briefly review the formalism for forecasting the rates of gravitationally lensed transient detections and phrase it as a model for the relative rate of gravitational lensing detections that conveniently side-steps messenger-specific technical details (§4a). We then apply this model simultaneously to all messengers and present an integrated view of relative detection rates across gravitationally lensed transient sources and the different messengers (§4b). This integrated view motivates the focus of the rest of this article on gravitationally lensed CBCs, beginning with a review of the available discovery channels (§4c).

We also introduce the term *golden object*, echoing how several breakthrough discoveries of individual objects have driven very significant scientific progress, in some cases over many decades, for example, GRB170817A/GW170817/AT2017gfo and the Hulse–Taylor pulsar. For convenience in this article, we define *golden objects* as gravitationally lensed sources for which multiple gravitational images are detected directly via many messengers, at least one of which is not electromagnetic. In the context of lensed CBCs—the main focus of this article—this would include a lensed BNS for which multiple images of the lensed merger are detected directly in GWs and more than one EM messenger.

### (a) A model for multi-messenger gravitational lensing rates

Previous works on the rates of gravitationally lensed transients [13,53–55,67,69,76,93–95,97,99,100,220] are based on an underlying framework that can be summarized as


(4.1) $$\begin{array}{lcr} & R_{\mathrm{lensed}}=\int d\Lambda \int dz\int d\mu \;\;\frac{d\tau }{d\mu }\;\frac{dV}{dz}\;\frac{R(\Lambda ,z)}{1+z}\;K(z)\;p_{\det}(\Lambda ,\mu ,z)\;, & \end{array}$$

where $R_{\mathrm{lensed}}$ is the number of gravitationally lensed object detections per unit time in the observer’s frame, Λ are the intrinsic source properties (e.g. luminosity, mass), z is the redshift of the sources, dV is the comoving volume element, R is the comoving rate density of the sources, K(z) describes how cosmological redshifting alters detectability (analogous to optical k-correction [222]) and $p_{\det}$ is the detection probability for a given messenger. For definiteness, equation (4.1) expresses the optical depth to gravitational lensing, τ, in terms of gravitational magnification, μ, for the reasons outlined in §3i.

The comoving rate density of sources is conveniently phrased as a separable function of redshift and intrinsic source properties, $R=R_{0}\;g(z)\;\phi (\Lambda |z)$, where $R_{0}$ is the local comoving rate density, g(z) describes redshift evolution and ϕ(Λ|z) represents the probability density function of CBCs, or luminosity function of optical sources. Uncertainties in these terms are the dominant sources of uncertainty in the *absolute* number of detectable lensed sources. In particular, the local comoving rate density and the redshift evolution of the sources are often not accurately known. Unknown redshift evolution is important because gravitational magnification enables sources to be detected at redshifts beyond those upon which models for R are based. Conversely, discovering gravitationally lensed sources at high redshift and/or well-defined non-detections can constrain the redshift evolution of the respective source populations.

**Figure 3.**
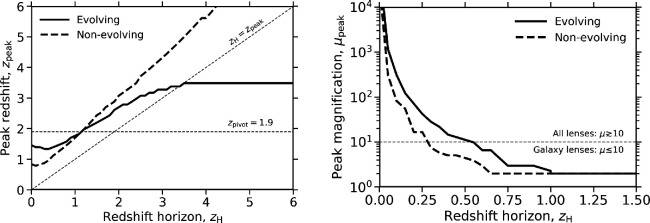
The peak of the redshift (left) and magnification (right) distributions of detectable lensed sources, as a function of redshift horizon, $z_{H}$, based on the model and assumptions described in §4a. The comoving rate density evolution of the ‘evolving’ population tracks the SFRD history of the Universe, as described in the text. In the evolving (more commonly used for forecasting) scenario, $z_{H}≃0.5$ is the approximate transition from detectable lensed sources being dominated by high-magnification lensing (μ≳10) to being dominated by low-magnification lensing (μ≲10).

Recent work on the detection rates of gravitationally lensed GW signals tends to focus on the *relative* rate of detection, i.e. the ratio of lensed detections to detections that are not lensed. This approach has the benefit that $R_{0}$ cancels, and uncertainties on the functional form of g and ϕ mainly impact on the redshift and magnification distribution of the detectable lensed populations (see below and figure 3). In what follows, we adopt typical (and benign) assumptions for g(z), namely non-evolving or evolution that tracks the evolution of the cosmic star formation rate density (SFRD) [223]. In the latter scenario, the SFRD peaks at a so-called pivot redshift of $z_{\mathrm{pivot}}=1.9$ and declines as a power law at lower and higher redshifts from that peak. While this model is not strictly relevant to the details of all source populations, it serves as a useful baseline for the overview presented here.

We write the relative rate of detection, ϱ, and the associated rates of detection for events which are lensed and not lensed as follows:


(4.2) $$\varrho \left(z_{H}\right)=\frac{R_{\text{lensed }}}{R_{not}}=\frac{\int_{z_{\min}}^{z_{\max}}dz\int_{\mu_{\min}(z)}^{\infty }d\mu \frac{d\tau }{d\mu }\frac{dV}{dz}\frac{g(z)}{1+z}K(z)}{\int_{z_{\min}}^{z_{H}}dz\frac{dV}{dz}\frac{g(z)}{1+z}K(z)}$$

where $\mu_{\min}(z)$ is the minimum gravitational magnification required to produce a detectable signal from a source/messenger combination, and (*Z*_min_, *Z*_max_) denotes the redshift range over which the respective detectors are sensitive to the different messengers. Also, $z_{H}$ are the redshift horizons for representative sources (§2, table 1) out to which they are detectable without assistance from gravitational magnification, at the signal-to-noise ratio (SNR) limit required for detection by the respective communities. In effect, for each source/messenger combination, we collapse Λ to a single parameter Λ and assign it a single value that corresponds to the minimum signal strength that is detectable at the relevant value of $z_{H}$. Finally, we assume K=(1+z), because this is relevant to band-limited detections [222]. However, the takeaway messages from this section are unchanged if we were to assume K=1.

To illustrate the sensitivity of the predicted lensed populations to the redshift horizon (as a combination of intrinsic source strength and detector sensitivity) and assumed redshift evolution of the source, we numerically integrate (equation (4.2)) and compute the peak of the predicted redshift and magnification distributions. The redshift distributions for lensed detections of evolving and non-evolving populations differ strongly (figure 3). For the evolving population, $z_{\mathrm{peak}}$ is tugged towards $z_{\mathrm{pivot}}$, and thus the detectable lensed population is dominated by sources at z≃1−3. In contrast, the $z_{\mathrm{peak}}$ for the non-evolving source population is always at $z_{\mathrm{peak}}>z_{H}$. For $z_{H}≲1$, an evolving population therefore peaks at higher redshift than a non-evolving population, and thus is more highly magnified. This behaviour reverses at $z_{H}≳1$; however, it is important to note that for these redshift horizons, detections of gravitationally lensed evolving and non-evolving source populations are dominated by low-magnification lensing events, i.e. μ≲10, and $\mu_{\mathrm{peak}}$ is dominated by the minimum magnification required for strong lensing. Therefore, for $z_{H}≳0.5$, multiply-imaged detections will be dominated by galaxy-scale lenses, while for $z_{H}≲0.5$, multiply-imaged detections will be distributed across the mass function of lenses including groups and clusters of galaxies.

### (b) Relative detection rates by messenger and source population

**Figure 4.**
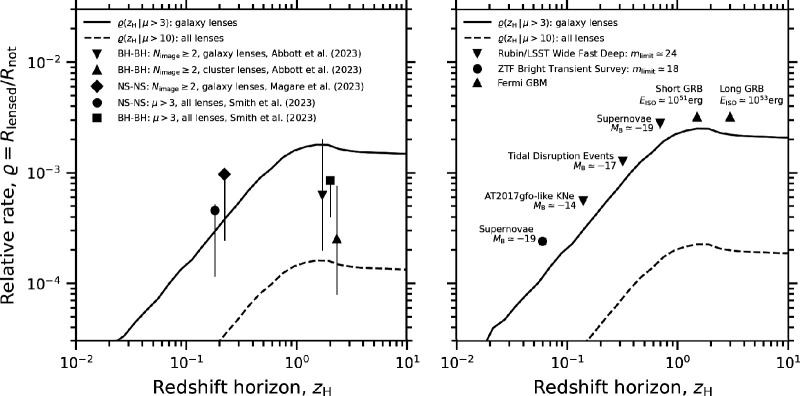
*Left*: The predicted relative rates of discovery of gravitationally lensed GW sources from a number of detailed studies (data points) overlaid on curves based on the multi-messenger model discussed in §4a. The upper (solid) and lower (dashed) curves bracket the range of threshold gravitational magnifications above which galaxy-scale lenses and all lenses (i.e. including massive galaxy clusters) are efficient at forming multiple images. The curves assume K=constant, as is relevant to GW detectors. *Right*: The typical redshift horizons out to which different EM sources can be detected without assistance from gravitational magnification ($z_{H}$, points), shown at an arbitrary offset above the curves, for clarity. The curves assume K∝(1+z) for simplicity, i.e. the k-correction relevant to an EM source that has a flat $S_{\nu }$ spectrum and detected photometrically. The difference between solid and dashed curves is the same as in the left panel. The expected relative rate of detection of gravitationally lensed images by the respective surveys can be read off from curves at the redshifts that correspond to each of the points.

The relative detection rate, ϱ, of gravitationally lensed images increases rapidly at $z_{H}<1$ before plateauing at one lensed detection per $≃10^{3}$ detections that are not lensed at $z_{H}≳1$ (figure 4). In the left panel, the model for $\varrho (z_{H})$ is consistent with several independent predictions for gravitationally lensed GW sources that are based on detailed calculations [13,69,76,93–97,99,224]. In summary, roughly one per thousand GW sources detected in the 2020s and 2030s is expected to be gravitationally lensed, independent of detector sensitivity. In the coming decade, the improving sensitivity of GW detectors combined with the relative lensing rate of $\varrho ≃10^{-3}$ will enable significant numbers of detections of gravitationally lensed GWs from LVK’s O5 onwards (table 1). The prospects are even stronger for future detectors, such as ET or CE, that are expected to detect $≃10^{5}$ GW sources per year [160,161], including lensing of other types of CBCs.

Redshift horizons lower than those shown in figure 4, i.e. $z_{H}<0.01$, correspond to source/messenger combinations that are not detectable beyond $D(z_{H})≃40\;\mathrm{Mpc}$ unless the signal is boosted by gravitational lensing. At $z_{H}<0.01$, the relative rate is very low, $\varrho <10^{-4}$, driven by the extreme gravitational magnification ($\mu ≳10^{6}$, i.e. beyond the upper limit on magnification that is typical of finite source effects [225]) required to detect a source at a typical redshift of z≃1−2 if the redshift horizon is $z_{H}<0.01$. Note that the local group is at lower redshifts still. Moreover, the cosmological volume interior to these redshifts is very small, rendering the number of detections of sources that are not lensed to be very small at $R_{not}\ll 1\;yr^{-1}$. For example, even next-generation GW detectors will only be sensitive to core-collapse supernovae within our own galaxy, and next-generation neutrino detectors are expected to be sensitive within the local group of galaxies. As alluded to in §2, this is the motivation for focusing this article on messengers from gravitationally lensed CBCs.

Turning to the right panel of figure 4, the relative detection rate of gravitationally lensed images is one per $≃10^{3}-10^{4}$ across EM messengers from sources discussed in this review. Therefore, as the number of detections that are not lensed approaches $R_{\mathrm{not}}≃10^{3}-10^{4}$, the detection of gravitationally lensed images becomes more likely. This is consistent with the detection of a few gravitationally lensed SNIa by the combination of iPTF and ZTF [10,11], the expectation that some of the $≃10^{4}$ GRBs that have been detected to date are in fact gravitationally lensed [164] and preparations to discover hundreds of gravitationally lensed supernovae with Rubin/LSST ([53–56,221] for example).

### (c) Pathways to multi-messenger gravitational lensing discovery

In general, discovery requires candidate gravitationally lensed signals to be selected from the many signals that are detected, as the trigger for follow-up analysis and observations. Efficient selection requires the lensed signal to be distinctive in some way relative to signals that are not lensed. Typically, this relies on gravitational magnification to make lensed sources appear to be brighter and closer than their true brightness and distance, and/or the detection of two or more signals that are consistent with being lensed images of a single source.

**Figure 5.**
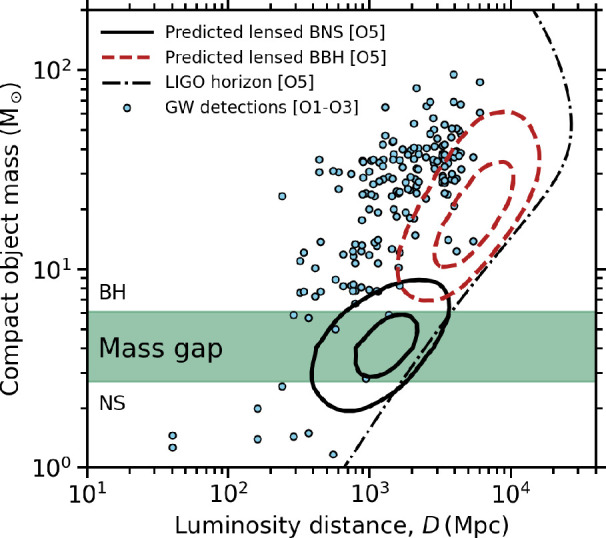
GW signals from gravitationally lensed BBHs during the fifth LVK run (as inferred in low latency, assuming μ=1) are predicted to overlap in mass with the bulk of the GW signals—compare red dashed contours with the detections from the first three runs. In contrast, GW signals from gravitationally lensed BNSs are predicted to be dominated by sources that appear in low latency to be located in the so-called ‘mass gap’ between neutron stars and stellar remnant BHs. This allows a more efficient selection of candidate-lensed BNS using magnification-based methods than for candidate-lensed BBHs. This figure is based on work published in [13,119].

To bring the focus to gravitationally lensed CBCs, we summarize some of the challenges involved in selecting candidate gravitationally lensed GW sources. First, the relative detection rate of $\varrho ≃10^{-3}$ (§4b) motivates assuming that GW detections are not gravitationally lensed unless strong evidence emerges to the contrary. The mass and distance of GW detections are both degenerate with lens magnification, and therefore, they appear brighter and closer than they really are, analogous to EM detections. However, the predicted masses that the LVK collaboration would infer in low latency (i.e. assuming μ=1) for gravitationally lensed GW sources have significant overlap with the range of masses of GW sources that (given that $\varrho ≃10^{-3}$) are unlikely to be lensed. For example, in LVK’s fifth run, based solely on the mass axis in figure 5, essentially every GW detection at $M≳2\;M_{⊙}$ could be regarded as a candidate gravitationally lensed GW source based on a magnification argument, and should, in principle, be included in dedicated lensing analyses to be confirmed/ruled out, such as was done in previous observing runs [76,226]. Note that the data points in figure 5 relate to GW sources detected in previous GW runs and are therefore subject to a horizon factor of ≃5× leftward (lower distance) than the O5 horizon that is shown.

Rapid *and* efficient identification of candidate gravitationally lensed GW sources is most critical for those sources that have transient EM counterparts, because both speed of EM ToO follow-up observations and suppression of false positives among the candidate-lensed GW sources are essential. The need for rapid follow-up, including the science case for detection of the first lensed kilonova image to arrive (§5c(i)), motivates a magnification-based selection of candidates if false positives can be adequately controlled. The putative ‘mass gap’ between the most massive NSs and the least massive BHs [162,227–231] is a promising region of parameter space in which to select candidate-lensed BNS mergers. The appeal is mainly empirical, in that this region of parameter space is sparsely populated, and not based on asserting that this region is empty of sources that are not lensed. The main strength of this discovery channel is the proven association of GWs, kilonovae and GRBs with BNS mergers, and thus the potential to discover *golden objects*. The challenges include the diversity of intrinsic properties of kilonovae, large GW sky localization uncertainties and the relative rarity of BNS mergers.

Most, and potentially all, BBH mergers are EM-dark, and hence, the emphasis on rapid identification of candidate-lensed BBH mergers among GW detections is less severe than for candidate-lensed BNS mergers. This, coupled with the significant overlap in the mass distributions of BBH mergers that are lensed and not lensed (figure 5), motivates a greater focus on selecting candidate-lensed BBH mergers for further investigation via image multiplicity. The main strength of this discovery channel is that BBH mergers are more numerous than BNS mergers among GW detections, and thus detection of the relevant GW signals by LVK is more likely. The challenges include the large GW sky localization uncertainties that will contain many gravitational lenses even after significant improvements in the sky localization derived from the joint posteriors of two GW detections. Nevertheless, magnification-based selection of candidate-lensed BBH sources is possible, for example in association with the follow-up ToO observations of massive BBH detections to search for AGN flare counterparts, following the candidate counterpart to GW190521 discussed by [121–124]. It is, however, noted that this focus is not exclusive; signatures of gravitational lensing may also be detected in individual GW detections, through waveform distortions resulting from microlensing or millilensing, or Type II images due to their negative parity.

**Figure 6.**
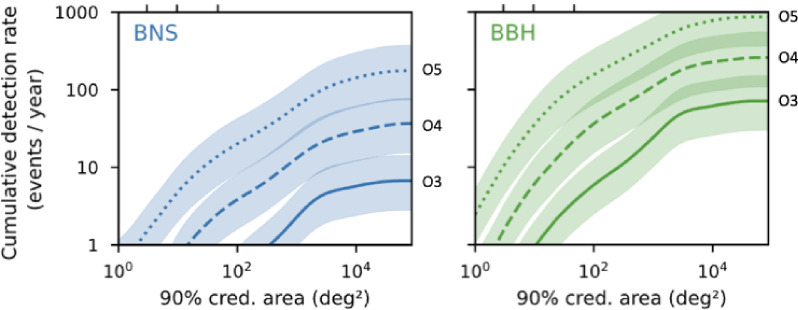
This figure is adapted from the figure available at https://emfollow.docs.ligo.org/userguide/capabilities.html, based on [232]. It shows the predicted cumulative distributions of sky localization uncertainties of GW detections by LVK through to their fifth run. Independent of run or source type, ≃10% of detections will be localized to better than *Ω* ≃100 degree2 precision. Improvements on this await the extension of the GW detector network, via LIGO India [233]. We also note that in the case where several lensed images are detected, major improvements in the sky localization uncertainties are possible [18].

Before moving on to discuss the channels introduced above in more detail, we provide further context on GW sky localization uncertainties in figure 6. As the sensitivity of the current GW detector network improves towards O5, the fraction of GW detections with sky localizations of $\Omega_{90}≲100\;{degree}^{2}$ remains at around 10%. This fraction will increase significantly when the planned LIGO-India detector comes online [233]. The size of the GW sky localization uncertainties is key to the synergies between GW and EM messengers, both for efficient use of telescope time to follow up GW sources and for efficient comparison with EM-based catalogues of known gravitational lenses. It is also important to note that the detection of multiple GW signals from a gravitationally lensed CBC merger helps to reduce the sky localization uncertainties considerably.

#### (i) Gravitationally lensed binary neutron star mergers

**Figure 7.**
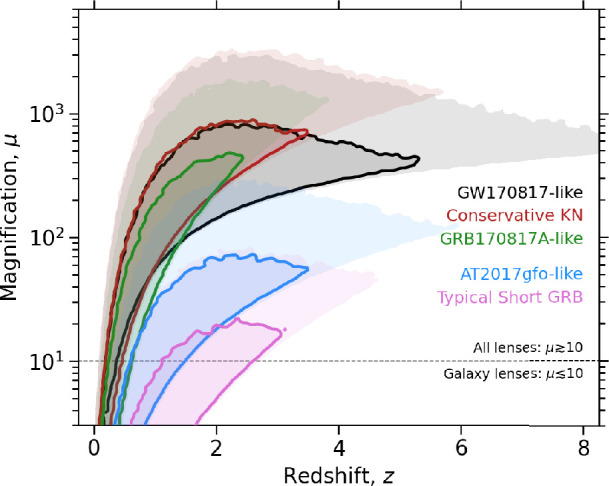
Magnification-redshift distributions of messengers from gravitationally lensed BNS mergers, based on table 1, equation (4.2) and §4a. Contours enclose approximately 90% of the predicted lensed detections, and the shaded areas extend to approximately 99% to visualize the tails of the respective distributions, as explained in §4c(i).

The detection of multiple messengers from a BNS merger in 2017 (GRB170817A, GW170817, AT2017gfo), combined with current/imminent detector sensitivities, has opened up the exciting prospect of detecting a gravitationally lensed CBC via multiple messengers. To give a concrete example, in figure 7, we show the location of detectable messengers from a gravitationally lensed BNS merger in LVK’s fifth run, based on the multi-messenger lensing model described in §4a, and assuming the LVK A+, LSST ToO and *Fermi*/GBM sensitivities listed in table 1. For each messenger, the lower edge of the respective contour represents a hard detection limit based on the respective horizons and the implied gravitational magnification required for detection. The detectable messengers have a tail to high magnification, as is apparent from the extension of pale shaded regions beyond their respective contours.

In the context of initial discovery via GWs (black contour), the key takeaway from figure 7 is that none of the other distributions are peaking/extending to higher magnifications than the black contour and pale grey shading. The EM instrument sensitivities are therefore well-matched to detecting the EM counterpart to an LVK detection of a gravitationally lensed BNS signal. Importantly, this is not strongly dependent on the details of the EM signals because the red and green contours that overlap well with the black GW contour are based on conservative assumptions about the brightness of EM signals. The red contour assumes that the kilonova counterpart is redder and fainter than AT2017gfo, following the ‘conservative’ model discussed by [13,119,153]. Equally, the green contour assumes that the GRB counterpart is fainter than a typical short GRB, for example, due to being viewed off-axis, as was GRB170817A. The blue (AT2017gfo-like kilonova) and pink contours (typical short GRB) correspond to brighter EM scenarios in which a lensed BNS merger that is detected by LVK in GWs would be detectable as a kilonova and short GRB, albeit in the respective high-magnification tails.

**Figure 8.**
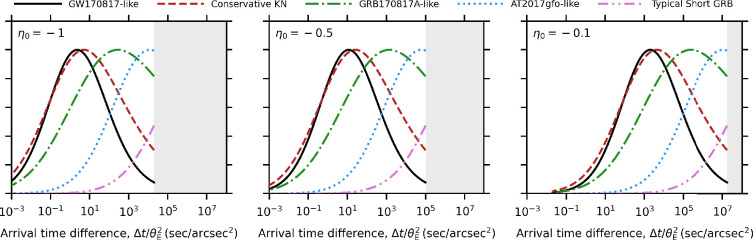
Arrival time difference distributions for the five messenger/instrument combinations shown in figure 7, normalized to an Einstein radius of $\theta_{E}=1\;\mathrm{arcsec}$, based on combining the magnification distributions shown in that figure with equation (3.13), for lens density profiles that are steep ($\eta_{0}=-1$), intermediate ($\eta_{0}=-0.5$) and flat ($\eta_{0}=-1$) at the mid-point between fold image pairs (§3f,g). The grey shaded region in each panel indicates the region in which μ<10, i.e. where lenses with flatter density profiles tend to be less efficient at forming multiple images (§3g). The distributions are all normalized to the same arbitrary peak value. The overlaps of the arrival time distributions shown in this figure reflect the overlapping distributions in figure 7.

Identification of GW signals from candidate gravitationally lensed BNS can be based on identifying sources that have a high probability of comprising one or more compact objects with mass consistent with $3<M<5\;M_{⊙}$ [13,14,117]. This selection, based on the information released with low latency by LVK, is also the baseline for the current planning of Rubin/LSST ToO follow-up of candidate gravitationally lensed BNS [119]. Clearly, a joint magnification plus multiplicity selection would be extremely powerful if the arrival time difference between two lensed GW signals is Δt≲1h, as highlighted by [13,99]. The short arrival time differences associated with lensed BNS mergers are a direct consequence of the relatively large magnifications required to detect them. For example, the arrival time difference between a fold image pair formed by a galaxy-scale lens (likely part of a quad image configuration) can be typically as short as a second and typically reach a day for a very flat cluster-scale lens (figure 8). The shorter arrival time differences for lensed BNS mergers therefore have the potential to probe the Eikonal optics regime (§§3d and 5b(i)).

Additional information from the GW data also has significant potential to suppress false positives when selecting candidate-lensed GW signals. For example, the mass ratio of the CBC and its detector-frame chirp mass are both invariant to gravitational lensing, and therefore are well-suited to improving the selection of candidates, if available. Detection of GW signals that appear to emanate from the mass gap and that also contain signatures of tidal deformability of the compact objects involved in the merger that are consistent with them being NSs would also add further weight to a magnification-based mass gap selection [234]. This enhanced method likely awaits next-generation GW detectors because measurements of tidal deformability of GW sources are rather poorly constrained with current GW detectors ([162,235] for example).

We also consider the scenario of EM-led detection of gravitationally lensed BNSs. Again, to give concrete examples, if this was based on multiple detections of a typical short GRB and/or a gravitationally lensed AT2017gfo-like kilonova, then the corresponding GW signals are unlikely to be above the detection threshold of the LVK data. This can be seen in the blue and pink contours being below the black contour in figure 7. Therefore, if LVK was operating at the time of such a detection, then a sub-threshold search of the LVK data would probably be required to search for the GW signals, following similar approaches to sub-threshold GW searches on GRB detections [236]. A GRB-led approach also highlights that the initial GRB sky localization uncertainties can span thousands of $degree^{2}$. To succeed, GRB-led discovery would therefore require progress on rapid identification of candidate-lensed GRBs (via multiplicity), and then rapid localization via their afterglow and/or kilonova emission [119,164].

#### (ii) Gravitationally lensed dark binaries

To date, the number of GW signals detected from BBH mergers outnumbers those from BNS mergers by a factor of ≃50 [4,158,237], and the rate of lensed GW detections is also expected to follow this pattern, assuming the two types of mergers follow the relative lensing rates (valid only for next-generation detectors). The detection rate of BBH mergers continues to grow and indicates that LVK will be capable of detecting a few lensed BBH mergers per year during their fifth run in the late 2020s [13,93,94,96–98,100]. Various tools and pipelines have been developed in recent years to analyse and identify lensed candidates in LVK GW data, though no conclusive evidence for lensing has been found so far [70,76,78,79]. For single, standalone GW events, searches for lensing signatures such as Type II images [87,172–174], micro- and millilensing [198,238] are conducted. For multiple images occupying similar regions of the GW parameter space, we can analyse the pairs, triplets or quadruplets against the chance of coincidental parameter match [82,85,87,174]. This is particularly effective for regions of the parameter space that are less densely populated, such as very high-mass events. While the risk of coincidental association between multiple candidate images increases with the number of detected GW events [97,239], introducing ‘time-delay windows’ (i.e. limiting the time window within which events are paired up together in lensing searches), according to predicted time-delay distributions from gravitational lenses, significantly reduces the false-alarm probability [69,97,240,241].

BBH mergers are not typically expected to be accompanied by direct EM counterparts—although see [120–124] for intriguing candidates, of which we discuss the AGN disk scenario in more detail as follows. The EM counterparts to NSBH mergers are expected to be fainter than counterparts to BNS mergers ([154] for example).

**Figure 9.**
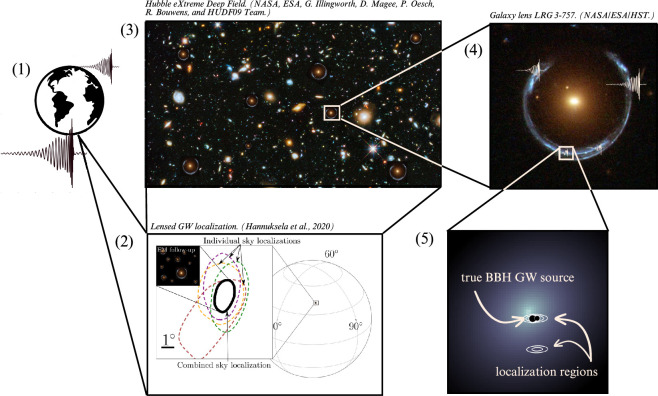
A schematic for the steps to localize a dark-lensed binary merger. (1) Lensed GW images are detected by the ground-based LVK observatories. (2) The sky localizations from the multiple identified images can be analysed jointly to reduce the final sky localization region [15]. (3) The joint sky region can be cross-matched with gravitational lens catalogues from LSST, *Euclid* and their contemporaries. (Edited from NASA, ESA, Illingworth, Magee, Oesch, Bouwens and HUDF09 team.) (4) The candidate lenses are individually analysed and reconstructed to test their match to the GW images. (Edited from: NASA/ESA/HST.) (5) If a gravitationally lensed galaxy from the EM lens catalogues stands out as a distinctly high-ranked candidate host of the dark-lensed CBC merger, the CBC can then be localized accurately in the source plane.

Host identification is a challenge for all CBCs without an identified EM counterpart (figure 9). However, with strong lensing, we obtain multiple images of the same GW event. If each of these images is strong enough to be initially detected as if they were independent GW observations, they each come with their own O(10−1000) degree${,}^{2}$ sky localization [242] that can be jointly analysed to reduce the localization to O(10) degree${,}^{2}$ for double- and triple-lensed GWs, and O(1) degree${,}^{2}$ for quadruplets [18,86,243]. We can also first do ‘dark lens reconstruction’ by using the properties of the lensed GW signals themselves to narrow down the parameter spaces of the lens directly, though this remains subject to degeneracies, in particular for axially asymmetric lens models [205,244]. This lets us narrow down the list of candidate lenses and hosts in the sky region, and the full lens reconstruction of the candidate lens profiles can then test if a particular lens model created the observed GW event [16–18,238,243,244]. When the sky localization region is sufficiently constrained, the lens is uniquely identifiable and the lensed host galaxy is bright enough, the host galaxy can be identified in up to about 30% of cases for quadruply-lensed GWs [16]. With upcoming detectors, the rate of lensed GWs is forecasted to increase, and we will be able to observe multiple lensed events each year, giving us information about the broader population of GW hosts.

From the GW side, the principal challenges remain around instrument sensitivity. The ability to detect as many of the lensed GW images as possible also carries a dependence on GW detector run length and duty cycle, therefore reducing the efficiency of discovery [13]. Higher detector sensitivities can reduce the number of lensed images missed, which will assist in better constraining the lens parameters and the sky region, which is crucial for EM follow-up observations and cross-matching with EM-based lens catalogues. More detectors operating will also significantly improve sky localizations [233,245]. Dedicated methods to find weaker images, which would separately fall below the usual detection threshold, by leveraging information from one or more already detected images (referred to in the literature as ‘targeted sub-threshold searches’) [71,74,81,246,247] can also help to increase the multiplicity of detected lensed systems. The sensitivity of such searches can, in turn, be improved by obtaining better constraints from lens models or lists of candidate lenses.

Improvements in the success rate of host galaxy identification will also come from deeper and more complete catalogues of gravitational lenses from EM surveys such as Rubin/LSST and *Euclid* [248,249] and improved empirical understanding of the covariance of lens density, structure, mass and image multiplicity (§3g). Both survey sensitivity and sub-arcsec second angular resolution are critical for GW host identification. The former lets us maximize the number of lenses identified, while the second provides enough detail about lenses for initial reconstructions to narrow down the candidate lists as much as possible. Should the initial resolution not be high enough, or the lens not unique enough, to identify a single host candidate distinctly, the top-ranked candidates would need higher-resolution dedicated follow-up observations, for example with the *Hubble Space Telescope*, *James Webb Space Telescope* and 30 m class telescopes.

Another possible avenue comes directly from the example of GW190521, a high-mass BBH GW source [250,251]. This event prompted a great deal of interest not only due to its high mass but also due to its possible association with an AGN flare [121–124]. When a BBH merger occurs in an AGN disk, the merger can cause a shock inside the gas disk that results in an observable flare [252–254]. In this particular case, the AGN flare’s association with the BBH merger remains uncertain [122–124]. Detecting a lensed AGN flare associated with an unusually high-mass BBH event could thus be considered a direct observable counterpart to a lensed BBH [119]. Furthermore, since a substantial fraction of AGN-disk BBHs are expected to be strongly lensed by the AGN supermassive BH, the non-detection of strong lensing can place constraints on the fraction of BBHs formed in AGN disks [125].

## 5. Multi-messenger gravitational lensing science

This section describes many of the science cases for multi-messenger gravitational lensing, organized under those relating to the nature of gravity (§5a), cosmology (§5b) and the physics of the source populations (§5c). Each science case includes a summary of the key challenges and progress that is required in the next 3−5 years.

### (a) The nature of gravity

As direct manifestations of the space-time metric, it is not a surprise that GWs offer new tools with which to probe directly the nature of gravity [255,256]. Gravitationally lensed GWs and EM counterparts expand and enhance these tools, thanks to the detection of multiple magnified copies of multi-messenger signals offset in time from each other, and the greater distances over which lensed signals typically travel relative to typical signals that are not lensed.

Deviations from GR that affect large cosmological scales are also a highly studied probe of the nature of dark energy. If such deviations exist, GWs should pass through the modified gravitational regime on their way from the source to our detectors, resulting in changes to the amplitude and phase evolution of GWs. Gravitational lensing can play a key role in revealing some of these changes; hence, the detection of a multi-messenger lensing event could offer new opportunities to pin down or rule out causes of cosmic acceleration. However, it is important to note that both lensing and departures from GR share some common phenomena. This could lead to the possibility of searches for either effect having false positives caused by the other. For example, see [257] for a broader review of possible sources of false positives in searches for deviations from GR and [258,259] for specific investigations for lensing and deviations from GR. Such false positive systematics must be carefully modelled.

Some common effects of cosmological modified gravity theories on GW propagation, that are discussed below, can be represented schematically as follows [260]:


(5.1) $$\begin{array}{lcr} & h_{ij}^{\prime \prime }+[2+\nu (z)]Hh_{ij}^{\prime }+\left[c_{T}^{2}(z)k^{2}+a^{2}m_{g}^{2}\right]h_{ij}=a^{2}\Gamma (z)\gamma_{ij}, & \end{array}$$

where primes denote derivatives with respect to conformal time, $H=a^{\prime }/a$ is the conformal Hubble factor, $h_{ij}$ represents either the plus or cross GW polarization and $\gamma_{ij}$ is a transverse-traceless tensor. $c_{T}^{2}(z)$ encodes the speed of propagation of GWs. The terms ν(z), $m_{g}^{2}$ and Γ(z) all represent new phenomenology [260]; the standard GR propagation equation on a Friedmann–Robertson–Walker metric is recovered in the limit ν(z), $m_{g}^{2},\;\Gamma =0$ and $c_{T}^{2}=1$.

Regarding the physical interpretation of equation (5.1), ν(z) is sometimes referred to as a ‘GW friction’ term, as it affects the rate of change of the GW amplitude as it propagates. In scalar–tensor gravity theories—the largest, simplest class of models—ν(z) is related to the time derivative of the gravitational coupling. This is equivalent to the rate of change of the effective Planck mass or gravitational constant. Meanwhile, $m_{g}^{2}$ represents the mass of the graviton; in GR, gravitons are massless, but they can become massive in other theories [261]. Finally, Γ(z) can be thought of as a source term for GWs; in GR, GWs are unsourced once they leave the region of their parent CBC. However, in some bimetric gravity theories, there can be interactions between the ‘normal’ metric $g_{\mu \nu }$ and a second tensor field, which acts to source the GWs as they propagate [262].

Apart from the graviton mass, a constant, the non-standard terms in equation (5.1) are all functions of redshift. For a specific modified gravity model, this redshift dependence can be computed directly from the gravitational Lagrangian. While, in principle, any functional form is possible; in practice, the time-dependence of $c_{T}^{2}(z)$, ν(z) and Γ(z) is often shaped by the hypothesis that deviations from GR should be responsible for late-time cosmic acceleration. That is, most modified gravity models are designed to leave the early universe unaffected and only deviate from GR at late times (say z≲2). That behaviour will be carried over to deviations from GR in equation (5.1). With this expectation in hand, phenomenological parameterizations of $c_{T}^{2}(z)$ and ν(z) have been widely investigated [263,264].

In reality, equation (5.1) must be corrected to account for perturbations in the gravitational field sourced by matter density fluctuations. These will source lensing and gravitational redshift effects for the propagating GW. The amplitude of these perturbations themselves can depend on the theory of gravity; indeed, this is one place where ‘screening’ effects may show up. Screening is a set of mechanisms by which modified gravity theories reduce to GR in particular environments: typically, highly dense perturbations will be screened (behave like GR), and linear perturbations will be unscreened (deviate from GR). The discussion of screening effects goes beyond the scope of the present work; see [265] for a comprehensive review.

#### (i) The first detection of gravitational lensing of gravitational waves as a test of GR

The first convincing detection of gravitational lensing of GW signals will itself be a first-of-a-kind test of GR since this theory predicts that GWs travel along geodesics and hence are gravitationally lensed as they traverse a gravitational field [27]. The detection of a lensed GW will confirm this property. From a multi-messenger perspective, in the absence of a direct counterpart, EM information such as comprehensive catalogues of gravitational lenses from surveys such as LSST and *Euclid* could provide additional support for candidate-lensed GW signals that are found by GW lensing searches by matching them with their lensed host galaxy ([70,76,78] for example). An initial proof-of-concept of such catalogue matching was, for instance, performed in [79] for some of the ultimately discarded GW lensing candidates from the third GW run. Should a lensed GW detection be accompanied by lensed EM counterparts (*golden objects*), we can take this a step further by testing whether GWs travel on *null* geodesics, i.e. whether they propagate at the speed of light.

#### (ii) Constraining the relative speed of messengers beyond GRB170817A/GW170817

In GR, both EM radiation and GWs are massless and should propagate at the same speed ($c_{T}^{2}=1$ in equation (5.1)). However, in some theories, the graviton can have a mass, and thus GWs can travel at a speed that is different from EM radiation. Any difference in speed can be measured when GW and EM signals are detected from the same source, known as *bright sirens*. However, a potentially confounding factor is that in a wave optics regime, GWs can sometimes *appear* to be travelling superluminally, due to distortions of the waveform [266].

The tightest constraints on $c_{T}$ are obtained from the most accurately measured arrival times, namely for GW and GRB signals. For GRB170817A/GW170817, the relative difference was constrained to be between $-3\times 10^{-15}$ and $7\times 10^{-16}$ [6,267–269], thus ruling out many alternative gravity theories. The dominant uncertainty in the analysis of GRB170817A/GW170817 is the GRB physics, i.e. the unknown details of the physics of GRB jet launching that can introduce a physical delay between GW and GRB emission that is not related to their speed [6].

Multi-messenger gravitational lensing offers a complementary method that side-steps the systematic uncertainty relating to the GRB physics. For each image of a lensed CBC detected in GWs and GRBs, one can measure the delay between the GRB and GW signals. Now, rather than analysing these delays individually, one can take the difference between them, thus eliminating the dependency on the GRB physics—namely the delay between GW and GRB emission—because it is the same for both lensed images. In this way, multi-messenger constraints on the relative speed of messengers, and in turn on the mass of the graviton, and possibly on the total neutrino mass, can be pushed to a new level [107–109].

An additional feature is that a joint GRB/GW detection will place a bound on the propagation speed of GWs at a much higher redshift (*z*≃1–2) than GW170817 (located at *z*≃0.01). While in GR, $c_{T}$ is a constant; in a modified gravity theory, its value can vary according to the cosmological evolution of (say) a scalar field or dark energy EoS. As such, bounding $c_{T}$ at higher redshifts has additional importance when constraining deviations from GR.

#### (iii) Probing GW propagation with gravitationally magnified sources

The new phenomenology introduced by modified gravity effects may be a function of redshift, as outlined in equation (5.1). Moreover, to be good dark energy candidates, most extensions of GR are constructed to reduce to GR at high redshifts (z≳2). Thus, as we noted above when discussing bounds on the GW propagation speed, probes that are capable of reaching the distant universe are particularly constraining. Detecting modified GW propagation as a function of redshift is challenging with solely GW data because redshift is not constrained directly by the GW data. Information about the redshift of a GW event is obtained from the set of siren techniques that were originally developed to measure $H_{0}$ from GW data [263,267,270–273]. We have already introduced bright sirens above [274], but let us note here that there exist two further techniques appropriate in the absence of EM counterparts, known as spectral sirens [275] and dark sirens [276–278].

Whether bright or dark, gravitationally lensed GWs are powerful probes of GW propagation [279–281]. Detecting multiple lensed copies of a GW event will enable tighter constraints on both the source parameters and the functions that describe modifications of GR, $\nu (z),\;m_{g}^{2},\;\Gamma (z)$ and $c_{T}^{2}(z)$, that appear in equation (5.1). Moreover, the lens magnification will allow the detection of distant systems which would otherwise not be detected, boosting our distance reach as motivated above. This will be most pronounced for gravitationally lensed BNS because they are expected to be more highly magnified than gravitationally lensed BBH [13,99]. However, even in the absence of an EM counterpart, a convincing gravitationally lensed GW source can benefit from other lensing information: identification of a plausible gravitationally lensed host galaxy consistent with the lensed GW signal will give direct access to the redshift of the GW source [15,16].

Maximal exploitation of a multi-messenger lensed event and forecast constraints with upcoming data is an ongoing area of study. Further work is needed to understand exactly how GWs propagate around a lens outside of GR (e.g. if the graviton has a mass) and how this would affect observables such as time delays and magnification ratios. All the subtleties of lens modelling in GR must be folded in on top of this (e.g. the precise location of the source within the host galaxy), and their degeneracy with modified gravity parameters investigated.

#### (iv) A step change in gravitationally lensed GW polarization constraints

GR predicts two GW polarization modes (+,×), in contrast to alternative theories of gravity that may predict up to six modes. Detecting polarization modes individually depends sensitively on the number of GW detectors because the GW signal at each detector is a linear combination of the GW polarizations, which depends on the sky location of the source relative to the detector. Due to the limited sensitivity of the present detector network, the current state-of-the-art employs simplified hypotheses as alternatives to GR. That is, the alternative hypothesis assumes that the polarizations contain only scalar modes or only vector modes (no tensor modes). These analyses have concluded that the tensor-only hypothesis is preferred over scalar-only or vector-only hypotheses [282–285].

Robust detection of multiple images from a gravitationally lensed GW source would at least double the number of GW signals available for polarization measurements of that source, with each lensed image of that GW source containing a different linear combination of the polarizations. This is because the lensed GW signals arrive at Earth at different times that are independent of the rotation of the Earth, and thus independent of the orientation of the detectors to the respective signals. This ≥2-fold increase in the number of signals therefore dramatically improves our ability to distinguish between the polarizations, simply by boosting the number of GW detectors from the four currently available to at least eight [113,286].

Methodologies need to be developed to extract the individual polarizations from lensed GW signals in a model-agnostic way to test GR efficiently. In the context of multi-messenger gravitational lensing, it is important to recognize that the best current GW polarization constraints come from the multi-messenger detection of GRB170817A/GW170817/AT2017gfo [285]. This is because the sub-arcsecond localization of the GW source, derived from the EM detection, delivers precise and accurate constraints on the GW detector responses (antenna pattern functions) to the GW polarizations. A *golden object* discovery would therefore facilitate constraining the additional polarization modes to the next level.

Some theories of gravity also predict novel birefringence phenomena for lensed GWs, whereby each GW polarization mode is deflected differently by a lens, leading to a net time delay between them [287–289]. Such modifications could be tightly constrained with a multi-messenger lensing event, although they could also imprint deviations that distort the waveforms themselves. On the one hand, detection of birefringence would violate GR, and on the other hand, strict limits on birefringence would constrain beyond GR theories. Treating this effect phenomenologically [290], found no significant deviation from GR using the latest catalogue of GW events (GWTC-3), and in turn constrained the birefringence probability and parameters of alternative theories of gravity. These constraints will get better as the number of detections increases. In addition to birefringence, other wave-optics phenomena provide a smoking gun for deviations from GR. Novel gravitational interactions also produce GW dispersion (frequency-dependent phase corrections) on the +,× and additional fields [291], and apparent polarizations distinct from GR [292]. Diffraction can provide even further tests of GR through frequency-dependent modulations of the amplitude [293,294].

### (b) Cosmology

Gravitational lensing is a powerful and well-established probe of cosmology, including the expansion of the Universe [35,295] and both the nature of DM and the structure and content of DM halos [183,296,297]. Measuring cosmic expansion with gravitationally lensed transient and variable sources—so-called time-delay cosmography—is central to resolving the tension between measurements of $H_{0}$ that are based on the distance ladder and on the cosmic microwave background [298]. The sensitivity of gravitational lensing signals to all matter, regardless of its nature, bestows upon lensing a central role in the quest to constrain DM. GWs and their EM counterparts—in the absence of lensing—are also emerging as valuable tools for cosmology, including as dark and bright standard sirens [270,274,276,277,299–302].

Multi-messenger gravitational lensing expands and enhances the cosmological applications of gravitational lensing in several important ways. First, the timing accuracy of GW, GRB and FRB instruments promises to push time-delay cosmography into a new regime of ultra-precise arrival time difference measurements. Second, a *golden object* will enable joint constraints on $H_{0}$ from multiple detections of the same bright standard siren and multi-messenger time-delay cosmography. Third, the ultra-precise timing of GW instruments in the wave optics limit and ultra-precise localization of optical detectors in the geometric optics limit are highly complementary for probing DM and the structure of DM halos.

#### (i) Multi-messenger time-delay cosmography

Multi-messenger gravitational lensing is an exciting new channel for time-delay cosmography, with the GW signal replacing the EM signal to measure the arrival time difference [110,303–306]. GW instruments have a timing accuracy of $approximately\;10^{-3}\;s$, which together with the well-understood GW waveforms for CBCs enables an arrival time difference measurement with an uncertainty of $approximately\;10^{-3}\;s$. For comparison, the most precise EM-based arrival time difference measurements to date have an uncertainty of ≃1day, reflecting the cadence of optical observations and optical brightness fluctuations [34] (table 1).

Gravitationally lensed GRBs (§5c(ii)) and FRBs (whether or not associated with a merger; §5c(iv)) would also yield a dramatic gain in the precision of the arrival time difference measurement relative to lensed quasars and supernovae, thanks to the sub-second timing accuracy of gamma-ray and radio instruments. Therefore, gravitationally lensed GW, GRB and FRB signals are all in the regime of ultra-precise arrival time difference measurements. To give a concrete example, the discovery of a multiply-imaged GRB that is localized to its gravitationally lensed host galaxy via its lensed afterglow emission would unlock ultra-precise time-delay cosmography.

In this ultra-precise arrival time difference regime, other uncertainties will dominate. Statistically, the relative astrometric precision of the arriving images will likely dominate [307]. For example, an arrival time difference precision of less than 1 s corresponds to a displacement of an image of order less than 10^−4^ mas, which is several orders of magnitude below the best possible astrometric constraints that would be achievable with space-based optical/IR follow-up observations of an EM-bright gravitationally lensed CBC such as a gravitationally lensed BNS merger. However, the ultra-precise time-delay measurements, in particular when measured in a quadruply-lensed system, can add significant constraints also on the lens model and the expected position of the images, thus mitigating, at least in part, the astrometric uncertainties [308]. For EM-dark gravitationally lensed mergers, such as BBH, accurate astrometry for time-delay cosmography will again rely on EM observations, for example via identifying the plausible handful of EM-detected gravitationally lensed host galaxies located within the joint GW-based sky localization of a candidate gravitationally lensed BBH [15,16].

A major systematic in time-delay cosmography is the modelling of the lens. The mass-sheet degeneracy (MSD) [203,204] is an important systematic uncertainty in time-delay cosmography and is relevant to all messengers (§3e). In the geometrical optics limit, the MSD cannot be broken with solely the lensing constraints upon which the $H_{0}$ inference relies. It can, however, be broken with measurements of the velocity dispersion of stars in the lens ([309] for example) or with weak lensing measurements [207]. The same or similar approaches are likely to be relevant to time-delay cosmography based on gravitationally lensed GW, GRB and FRB signals. In addition, the possibility of breaking the MSD in the Eikonal optics regime, using the beat pattern of two gravitationally lensed GW signals that overlap temporally, has been explored [200,208,209]. This is because the frequency-dependent distortions in the GW waveform encode more information about the lens model than just the time delay between the images. In brief, the MSD can potentially be broken with the GW data themselves if the arrival time difference is comparable with the duration of the GW signals (approximately1min in the case of BNS signals). The MSD may therefore be suppressed for multi-messenger gravitational lensing time-delay cosmography in the high-magnification regime that is typical for gravitationally lensed BNS mergers [13].

Further work in this area includes exploring and implementing optimal search strategies to identify multi-messenger lensing events that are well-suited to time-delay cosmography, given the predicted region of parameter space in which such lensed events will occur. Work is also required to investigate and develop optimal analysis methods to combine all/some of the messengers and to break the MSD. This work will also shape the requirements for follow-up observations of multi-messenger lensing events ahead of detections.

#### (ii) Gravitationally lensed standard sirens

The standard siren method of measuring $H_{0}$ combines GW-based luminosity distance measurements to CBCs with estimates/measurements of the redshift of CBC host galaxies to constrain the redshift–distance relation [270]. The first bright standard siren measurement was enabled by the multi-messenger discovery of GRB170817A/GW170817/AT2017 gfo [274]. The multi-messenger gravitational lensing analogue of this measurement would involve a standard siren measurement of $H_{0}$ for each of the images of a gravitationally lensed EM-bright CBC. The distance measurement for each image would be derived from the respective GW strain signal and corrected for gravitational magnification, while the redshift measurement would come from follow-up EM observations of the images of the lensed EM counterpart and/or lensed host galaxy. Such measurements of $H_{0}$ would extend the redshift reach of bright standard siren constraints from redshifts of z≲0.1 with LVK detections of EM-bright standard sirens that are not lensed to *z*≃1–2 for systems that are gravitationally lensed. While this science is relevant to *golden objects*, it is mainly reliant on GW and optical detections for the distance and redshift measurements, respectively.

EM-dark standard siren measurements have also been made using the growing catalogue of BBH mergers that LVK have detected [299,310–312]. It has also been demonstrated that gravitationally lensed EM-dark standard sirens can yield interesting constraints on $H_{0}$, by probabilistic ranking of plausible lensed host galaxies within the joint sky localization of pairs of candidate-lensed EM-dark GW signals [15,16,18,313]. A key advantage of this method over EM-dark standard sirens that are not lensed is that in the lensing case, the number of plausible host galaxies is *significantly* suppressed by the joint sky localization of two GW detections, the requirement that the host galaxy candidates must themselves be gravitationally lensed, and the galaxy lens must be able to reproduce the gravitational wave lensing observables.

Further work is required to build the Rubin/LSST and *Euclid* strong lens samples, as these will play a key role in this science for both EM-bright and EM-dark standard sirens. It is also important to develop methods to combine multi-messenger time-delay cosmography and gravitationally lensed standard sirens to optimize the synergy between these novel constraints on $H_{0}$.

For completeness, we also note that weak lensing of GWs can be a source of bias for the measurement of the Universe’s expansion. Gravitational potentials present on the GW travel path from source to observer will lead to additional magnification, which will bias the measured luminosity distance [314]. Moreover, magnified events are more likely to be detected, meaning it will worsen the bias [315–317]. Such biases can be accounted for as an additional source of noise in distance measurements [318]. For bright sirens, it can be shown that, in some cases, lensing can lead to bias larger than statistical uncertainty [314], also showing the importance of properly modifying the model of the lensing magnification probability density function [188]. For dark events, magnification can bias the source-frame chirp mass estimate as it would lead to a biased luminosity distance, and consequently, the redshift if no external observables can be used to alleviate the degeneracy [13,319,320].

#### (iii) The dark matter subhalo mass function

Numerous astronomical observations point to the matter content of the Universe being dominated by cold (non-relativistic) DM ([321–327] for example). However, on small length and mass scales, cold dark matter (CDM) faces a number of challenges, including the so-called ‘missing satellite’ problem ([328] and references therein). It has therefore been proposed that CDM may not be the correct picture, and there could be other kinds of DM such as fuzzy [329], interacting [330] or warm [331] DM.

Gravitational lensing is a well-established probe of the structure of the DM halos within which individual galaxies, groups and galaxy clusters are embedded. Much attention has focused on gravitational magnification and deflection, via gravitationally lensed quasar flux ratio anomalies, perturbations in the positions of lensed galaxies (astrometric anomalies) and the structure of galaxy cluster cores ([183,296,297,332,333] and references therein). DM substructure can also perturb the arrival time of signals from distant gravitationally lensed sources [334]. However, measurements of optical light curves are subject to intrinsic measurement uncertainties of ≃ 1 day (table 1). Such uncertainties likely swamp any arrival time perturbations induced by DM subhalos [335].

Joint multi-messenger probes of DM will enable a dramatic gain in the size of halos that can be probed, thanks to the synergy between time delay, astrometric and flux anomaly accuracy. Currently, optical strong lensing can detect DM halos down to masses of $M≳10^{7}-10^{8}\;M_{⊙}$ [336–338] via multiple separate images whose angular separation scales with lens mass M as $\Delta \theta \approx 2\;\theta_{E}\propto \sqrt{M}$, where $\theta_{E}$ is the Einstein radius. However, the angular resolution of optical telescopes limits the minimum detectable Δθ, which in turn limits the minimum detectable M. On the other hand, M can be obtained through the time delay. With sub-second timing accuracy, it will be possible to explore low-mass DM subhalos down to *M*≃10^5^-10^6^M⊙.

Gravitationally lensed GWs, GRBs and FRBs can provide arrival time difference measurements with sub-second precision. This ultra-precise arrival time difference regime is a promising new probe of DM subhalos. In brief, this new probe will exploit the synergy between optical/near-IR flux and astrometric anomalies and GW/GRB/FRB timing anomalies for EM-bright gravitationally lensed events ([109,335] for example). It is therefore well-suited to, but does not require, a *golden object*.

The idea of detecting lower mass lenses through time delays of the lensed images has previously been explored in the context of millilensing of GRBs. The principle is similar to strongly lensed GWs: detecting the repeated signals in the time domain instead of being limited by angular resolution [339,340]. However, since identical repeated GRB signals are difficult to identify [164], it has so far been challenging to test their lensed nature. This could be circumvented by detecting a lensed multi-messenger signal (of both strongly lensed GWs and a lensed GRB), where the time delays would coincide. Similar, albeit less accurate constraints on the sub-second time precision could be achieved for EM-dark gravitationally lensed events. This relies on probabilistic ranking of plausible host galaxies within the joint sky localization of candidate-lensed BBH candidates [15,16,18].

Further work is required to quantify the signatures in lensed GW signals arising from realistic populations of DM subhalos and properties such as their abundances, density profiles and radial distributions within the main lensing halo. We need to determine the probability of detecting such milli-lensed GW signals and whether the lens search pipelines will be sensitive to them. Many of the additional time-resolved, milli-lensed signals are likely to be demagnified and thus, possibly below the typical detection thresholds of lens search pipelines. For milli-lensed GW signals that are overlapping, it will also be important for future GW detectors to establish that the signals are actually lensed rather than a chance overlap of two unrelated GW events. Joint analysis in the optical and GW domains may provide suitable priors not only to help discover the milli-lensed GW signals but also to constrain the DM subhalo properties.

#### (iv) Microlensed GWs, the stellar mass function and compact object dark matter

**Figure 10.**
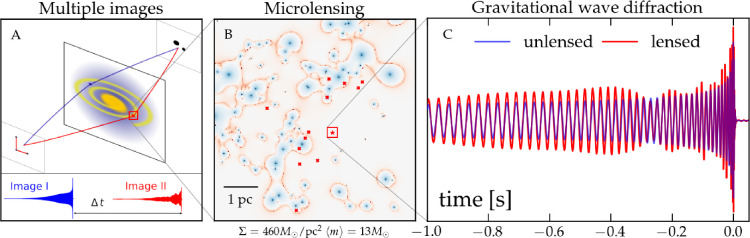
(A) A strongly lensed system produces two images of a GW source (lines), separated by an arrival time difference Δt (lower inset). (B) Image II encounters a large projected density of objects (dots) within the lens (with finite radius $10^{-4}\times$ the Einstein radius of the strong lens). Colour shows the magnification in the lens plane. The main image is shown as a star and microimages appear as crosses. (C) Microlensing produces a distinct modulation in the GW signal.

Microlensing can be used to characterize sources and the matter distribution of gravitational lenses on small scales, including stellar-origin objects and DM. Microlensing signatures have been detected from a variety of EM sources, and their correct treatment is essential to the robust interpretation of a wide range of lensed systems. Microlensing is an established probe of the structure of quasars [185], the mass distribution of stars and remnants in lens galaxies [185,341] and the size of supernovae [181]. Microlensing has also been used to constrain the abundance of compact DM objects using quasars [342] and caustic crossings of individual stars [95,343,344]. The sensitivity of EM microlensing is typically set by the finite size of the region emitting the flux that is microlensed (figure 10; [185]), rendering it challenging to constrain the mass function of microlenses [345].

Microlensing of GWs has not yet been detected [76,78]. This may stem from using waveform templates in GW search pipelines that do not incorporate signatures of lensing, resulting in a reduced detection efficiency for microlensed GWs [342]. The signature of GW microlensing is a modulation of the waveform (figure 10) caused by diffraction of the GWs by objects with masses of M≃$(8\pi Gf{)}^{-1}$≃$10\;M_{⊙}(1kHz/f)$, where f is the GW frequency [190]. However, in the regime considered here—microlensing of strongly lensed sources—the effective mass of the microlenses is rescaled by ∼μ, the magnification caused by the ‘macro lens’ responsible for producing multiple images [346]. On the analysis side, while microlensing by dense stellar fields within lenses is the most plausible origin of a detection [17,176–180,218,347], computational challenges in the wave optics regime have in the past restricted analysis to isolated point lens model, however, this has begun to change [79,83,348]. Microlensing of GWs is also sensitive to the small-scale DM distribution, which can be probed by microlensing of strongly lensed sources [349,350] and by diffraction by isolated lenses [190,191,194–197,351,352].

A clear advantage of multi-messenger microlensing is the synergy between two gravitational lensing regimes: EM microlensing in geometric optics and GW microlensing in wave optics, because together they provide a lever to investigate the mass of microlenses. For example, looking at existing EM studies, the quasar emission region sets the mass scale accessible to analysis of light curves without constraining the slope of the mass function [345]. On the other hand, GW microlensing is sensitive to heavier microlenses with M∝1/f. A multi-messenger source, such as an AGN binary in a lensed quasar, would constrain the stellar initial mass function (IMF) and stellar remnants simultaneously at low and high masses, surpassing the capacity that EM and GW have separately. On the other hand, joint analysis of microlensing of GW and kilonova signals from lensed BNS, even though probing essentially the same microlens population, will have distinct observational signatures in their respective domains. The former is frequency-dependent distortions and the latter will have time-dependent evolution of the microlensed light curve due to the increasing size of the kilonova. As a result, this method can produce stringent and unique constraints on the microlens population properties. In summary, while EM and GW microlensing have been studied separately to date, joint analyses will unveil exciting new opportunities.

Further work is required to develop the theory of microlensing to simultaneously account for the specific effects on GWs (frequency evolution) and EM signals (finite source size). Applications to data will require adaptation of computational tools for parameter estimation to include microlensing signatures, building on existing public codes [83,179,348,353–357]. Developing tools for dedicated searches of microlensed GWs will increase the sensitivity to events [342], especially when leveraging information on known EM transients with a potential association [358]. Incorporating information about microlensing in low-latency analyses can provide a rapid warning on a lensed GW, triggering follow-up searches for EM counterparts that may otherwise be lost. When estimating microlensing signatures and their rates, it is also important to revisit standard assumptions on the IMF and remnant formation channels, motivated by EM observations (not exclusively lensing) ([359–361] for example) and contemplate variations [179,342,362,363].

#### (v) Solar mass primordial black holes

A wide range of empirical constraints continues to permit some of the putative DM to be in the form of primordial black holes (PBH), whose mass function contains structures including a prominent peak at M≃$1\;M_{⊙}$ ([364] and references therein). It is therefore broadly accepted that convincing detection of so-called ‘solar mass BHs’ would be a smoking gun for the discovery of PBHs, because stellar evolution does not form BHs of this mass. Interpreting the GW sources detected by LVK in the context of PBH has therefore been an active field since the first direct detection ([365–370] for example).

Multi-messenger gravitational lensing is relevant to PBH because a gravitationally lensed merger of two solar mass PBH will occupy a similar region of the low latency (i.e. based on assuming μ=1) mass–distance parameter space as gravitationally lensed BNS (§4; [13]). In essence, gravitationally lensed solar-mass PBH mergers are a ‘false positive’ for gravitationally lensed BNS mergers. The main challenge in confirming the PBH interpretation will be whether the follow-up EM observations are sufficiently sensitive to rule out all possible EM signatures of a CBC that comprises one or more NS. Further work on this science case is therefore needed to enhance the selection methods of candidate gravitationally lensed CBCs and the design of follow-up ToO observations with the Vera C. Rubin Observatory. This will include detailed end-to-end modelling of the expected GW and EM signatures of gravitationally lensed EM-bright and EM-dark CBCs.

### (c) Physics of the source populations

Gravitational lensing is a well-established probe of the physics of distant source populations, including those that are only accessible with help from gravitational magnification. Multi-messenger gravitational lensing unlocks new opportunities, including novel probes of the physics of kilonovae and GRBs, the population of stellar remnant compact objects from which CBCs emanate, the nature of FRBs and their connection with other transient populations, the host galaxies of CBCs across cosmic time and the physics of core-collapse SNe.

#### (i) Kilonova physics

Constraining the EoS of dense nuclear matter is a fundamental question in nuclear physics. Observations of kilonovae provide constraints on the EoS in a region of parameter space that cannot be replicated in the laboratory because the NS interiors are among the only places in the Universe where macroscopic ‘cold’ matter exists at densities at least comparable with atomic nuclei. The observable properties of kilonovae are driven by the outcome of BNS mergers, which are all sensitive to the structure and EoS of the component NSs. These post-merger properties include the amount and composition of the material that is ejected, and whether the object that remains after the merger is a BH or short-lived NS.

Kilonovae are broadly classified as ‘red’ or ‘blue’ based on their observable properties. Red kilonovae are associated with ejecta with a low electron fraction ($Y_{e}$), which therefore produce lanthanides (elements with open f-shells) and have high opacity. Their high opacity prevents the escape of optical and UV photons, which scatter to lower energies through fluorescence before eventually escaping through opacity gaps in the IR [371]. Blue kilonovae are associated with high $Y_{e}$, Lanthanide-poor and low opacity ejecta. This lower opacity enables more optical photons to escape, leading to the term ‘blue’ [372]. The distribution of $Y_{e}$, and therefore the relative luminosity at blue and red wavelengths, is sensitive to the binary mass ratio and the EoS [373]. However, any blue kilonova emission is likely only detectable during the first day post-merger. Indeed, the ≃12 h delay between the detection of GW170817 and the Chilean sunset meant the rise of the optical emission from AT2017gfo was missed in the bluer bands. The early emission that was seen could also be explained by cooling of gas shock-heated by the GRB jet [374,375]. Crucially, only observations during the first few hours post-merger can differentiate these scenarios [376]. Despite the challenges, most studies agree that GW170817 showed evidence for multiple spatially distinct components with different $Y_{e}$ [12]. More detailed discussion of the ejecta properties inferred for GW170817 appears in [104,165] in this volume.

Gravitational lensing offers a unique window into the early evolution of a kilonova at blue wavelengths. Gravitationally lensed kilonova counterparts to gravitationally lensed BNS mergers are predicted to reside at *z*≃1–2 [13]. Optical searches, even in red bands, therefore probe rest-frame near-UV emission. Moreover, time dilation increases the effective window during which the early light curve can be detected, if searches are sufficiently sensitive. Rapid identification of the first image associated with a multiply-imaged kilonova/BNS with an arrival time difference of ≳1 day would enable targeted observations of the second image during the moments after merger. A single gravitationally lensed kilonova and BNS merger that is detected during the first days after merger (the only time during which detection is plausible) could therefore provide some of the best constraints on the rise and physical origin of the early UV emission. Combined with constraints from the GW signal, multi-messenger modelling can then be performed [153,154,377–379] to connect the pre-merger (e.g. mass ratio) and post-merger (e.g. blue ejecta) properties. The relation between these is determined by the neutron star EoS.

Detecting the early emission will require deep and rapid ToO imaging observations from the ground and space, that reach depths of AB≳25 over multiple nights to detect the lensed kilonova, ready for spectroscopic confirmation in the near-IR with the *James Webb Space Telescope* and 30 m class telescopes[13,14,104,119]. Progress is also needed to develop theoretical models to infer masses of r-process material from observations and to link observational signatures directly to the underlying EoS. Radiative transfer simulations can predict the expected kilonova signatures for merger ejecta compositions resulting from employing different theoretical EoS. However, many uncertainties still remain in merger simulations, r-process nucleosynthesis and atomic data and in kilonova radiative transfer modelling [165]. Additionally, there is the question of whether all the early blue emission is powered by radioactivity or if some or all of it results from the heating of polar ejecta by a long-lived jet [380]. With ongoing work in this direction, kilonova simulations could predict the timescales of the early blue component that would be measurable for different theoretical kilonova configurations, allowing observational constraints to be linked to the underlying EoS and r-process compositions synthesized.

#### (ii) Gamma-ray burst physics

As the most luminous explosions in nature, GRBs offer the ability to study lensed transients across the Universe and to probe arrival time differences as short as milliseconds (and hence lens masses down to $M<10^{4}\;M_{⊙}$). Multi-messenger detections offer a route to rapidly confirm lensing in GW sources (via multiple coincident GRBs) and to test fundamental physics using the speed of light and GWs (§5a(ii)). Independent of GW detection, multi-wavelength detection of lensed GRBs holds great promise for $H_{0}$ measurements, thanks to the timing accuracy of Gamma-ray instruments (§5b(i)). On the astrophysics side, lensed GRB detections are also an opportunity to better understand the properties of the GRBs themselves, in particular, because the relativistic outflows from GRBs may (or may not) have a structure on sufficiently small scales that lensed GRBs may be chromatic, and because multiple images of a single GRB could enable the multi-wavelength study of the emission from the earliest times in a similar vein to that discussed in §5c(i).

More than $10^{4}$ GRBs have been detected to date, and some of these have likely been lensed [164]. However, to date, no lensed GRBs have been confirmed, for example, by the identification of the lens, creating ambiguity over whether candidate-lensed GRBs are, for instance, caused by similar pulses within a single GRB or bona fide lensed GRBs. Multi-messenger detections of lensed GRBs are most likely to arise from GRBs that are associated with CBCs. Traditionally, this is the short-GRB population, although recent evidence also suggests that some long-GRBs may also arise from this channel [381,382].

The scientific impact of the discovery of lensed GRB arrival time differences in the seconds to hours range, for example as the counterpart to lensed GWs from a highly magnified lensed BNS merger [13], is enhanced by the fact that the second lensed image is likely to occur *while* observations of the field containing the first image are ongoing. Hence, rather than having only γ-ray data, the prompt emission can be observed in the X-ray, optical and plausibly even radio regimes. Constructing such broad-band SEDs of the prompt emission will be highly diagnostic and enhanced by, but not dependent on, a *golden object* discovery.

Lensing also offers a route to probing the angular structure of GRB jets. If GRB emission is anisotropic on small scales, then we may expect to observe multiple images which show chromatic variations. This both poses a challenge by creating uncertainty about whether temporally and spectrally identical bursts are an accurate route to identifying candidate-lensed GRBs, and an opportunity because it provides a direct route to determining structure in GRB jets on scales much smaller than the opening angle of the GRB, and hence, potentially discriminating between jet structure models. In particular, to distinguish between jets that are patchy, with hot and cold spots [383,384] or structured, with much stronger emission close to the axis [385,386].

Progress in this field will critically depend on the ability to promptly recognize lensed GRBs in close to real time, rather than identifying plausibly lensed events long after the burst, and afterglow are gone. Since GRBs are now detected by many different satellites (e.g. *Swift, Fermi, Einstein Probe, SVOM*) and the archive of old bursts is very large, the ability to rapidly correlate locations and light curves from different sources would greatly enhance the probability of correctly identifying lensed GRBs. The recent launches of both the *Einstein Probe* and *SVOM* should enhance the number of well-localized bursts in the coming years, increasing the possibility of rapid identification. However, their sky coverage is significantly less than *Fermi*/GBM, and thus rapid wide-field optical ToO follow-up from the ground, including with Rubin, will have a critical role to play for lensed GRBs that are identified in real-time [119].

The opportunity to probe jet structure also requires further investigation to understand quantitatively its impact on lensed GRB selection methods based on spectral similarity. For multi-messenger lensing, already running searches for coincidence between GRBs and GW detections are highly valuable, but should be extended to new missions to ensure events are not missed.

#### (iii) The mass function of stellar remnant compact objects

Robust constraints on the stellar remnant mass function are central to our understanding of stellar evolution and the physics of dense matter, including the formation channels of CBCs, the EoS of NSs and SN explosion mechanisms. Direct detections of GWs from CBCs have enabled significant progress in empirical constraints on the mass function in recent years, with detections of sources that comprise one or more compact objects in the putative ‘mass gaps’ attracting particular attention [162,231]. The lower gap is associated with an absence or paucity of compact objects with masses in the range $3≲M≲5\;M_{⊙}$, i.e. intermediate masses between the heaviest NSs and the lightest BHs [227–230]. The upper gap is associated with an absence or paucity in the range $50≲M≲120\;M_{⊙}$, and related to the fate of massive stars and the pair instability ([387] and references therein).

Gravitationally lensed CBCs detected via their GW emission can masquerade as residing in one of these mass gaps because lensed images of distant sources are gravitationally magnified, and thus the detections appear to originate from sources that are brighter and closer than the actual source. In particular, gravitational magnification increases the GW strain amplitude, which leads to underestimating the distance to the CBC that is inferred from the amplitude, and in turn to overestimating the source-frame frequency of the GW signal and hence overestimating the mass of the system ([319] for example). Thus, GW sources that are below a mass gap can appear to be in a mass gap if lensing is not accounted for in the data analysis. Recent GW detections include sources that—assuming no gravitational magnification, i.e. μ=1—populate both mass gaps [162,231]. Therefore, understanding the impact of gravitational magnification on the inferred masses and distances of GW sources is becoming critical to robust identification of real mass gap events that can be used for formation channel studies. Methods to break the magnification–distance degeneracy in the interpretation of the amplitude of GW strain signals ($A\propto \mu^{0.5}D^{-1}$) are of particular importance.

Multi-messenger gravitational lensing can break the magnification–distance degeneracy for candidate mass-gap GW detections, because detection of EM counterparts to GW sources is a proven way to measure the redshift of the source independent of the GW signal [24]. This is clearly relevant for GW sources that appear to be in the lower mass gap because the GW detector sensitivity for low mass sources (out to *z*≃0.2), the cosmological model and the physics of gravitational lensing combine to place the majority of gravitationally lensed BNS mergers in this region of parameter space—i.e. ‘in the lower mass gap’—if μ=1 is assumed [13] (figure 5). Deep and rapid ToO observations of GW sources that are initially placed in the mass gap are sensitive to a wide range of kilonova physics and can therefore probe both lensed BNS and not lensed interpretations of mass gap sources [14,117,119].

Multi-messenger gravitational lensing may also be relevant to the upper mass gap, motivated among others by the detection of a candidate AGN flare as a possible EM counterpart to GW190521 [121], and population models consistent with a fraction of BBH mergers forming via the AGN channel [388,389]. The dense environment in AGN accretion disks renders this channel prone to forming very massive BH binaries [390]. Therefore, EM follow-up observations of high mass BBH sources—i.e. tuned to search for AGN flare EM counterparts—are also well-matched to searching for AGN flare EM counterparts to gravitationally lensed BBH that are magnified into the upper mass gap [119].

Further work is needed to improve the selection of candidate-lensed GW sources from low latency information provided by LVK, with the overall aim of reducing the false positive rate within such selections and guiding the design of the follow-up observations. This will maximize the efficiency of the follow-up observations and optimize the range of EM counterpart physics to which they are sensitive. Multi-messenger simulations of the full range of detectable signatures of gravitationally lensed CBCs will be crucial, including to inform which properties of GW sources are most discriminating if released by LVK with low latency. Current lensing-motivated ideas for expanding such information include detector-frame chirp mass, mass ratio and the tidal deformation parameter [80,119,234].

#### (iv) The nature of fast radio bursts

FRBs are millisecond-duration radio transients that have intrigued the scientific community since their discovery in 2007 [126]. While the exact origin of FRBs remains uncertain, the comoving rate density of ≃$7\times 10^{4}$ Gpc${,}^{-1}$ yr${,}^{-1}$ for FRBs at energies above $10^{39}$ erg [391] far exceeds the rate of CBCs—10 to 1700 Gpc${,}^{-1}$ yr${,}^{-1}$ for BNS mergers [231]. CBCs therefore appear unlikely to account for the majority of the FRB population. Indeed, some FRBs are thought to be produced by magnetars, based on detections of bright radio bursts from the Galactic magnetar SGR, 1935+2154 in April 2020 [392,393], and those FRBs that repeat clearly are not associated with cataclysmic events [394,395]. Nevertheless, CBCs remain a credible origin for some one-off FRBs—i.e. the available data and theoretical models are consistent with FRBs originating from multiple progenitor channels [396–402].

Multi-messenger gravitational lensing is well-placed to probe whether there is a direct connection between FRBs and CBCs. Such a connection is currently difficult to establish due to the poor localization constraints of GWs and the unknown time delay between the occurrence of a CBC and associated GW emission and the emission of any radio burst. However, gravitational lensing provides a unique opportunity. If both an FRB and a GW signal from the same CBC event are gravitationally lensed, the time delays between the lensed images would be identical, offering a strong, unambiguous association between the two signals [403]. This would provide critical insight into whether CBCs can indeed produce some of the observed FRBs.

To date, progress in FRB observations has significantly improved our ability to detect and study these bursts. Interferometric techniques now allow for the precise localization of FRBs to their host galaxies [404], opening the possibility of identifying lensed FRBs. Moreover, many FRB surveys now store raw voltage data when bursts are detected, preserving the phase information of radio waves [405]. These data are crucial for identifying lensed copies of an FRB, even when propagation effects through the interstellar and intergalactic medium complicate signal detection. The complex spectro-temporal structures seen in many FRBs, especially at micro and nanosecond timescales, are intrinsic to the burst and can serve as a distinguishing feature to identify lensed copies [163].

In the upcoming years, progress in several areas will be essential for improving our prospects in multi-messenger gravitational lensing. Increasing the FRB detection rate and improving localization accuracy are key objectives for current FRB surveys [404,406–408], with the CHIME/FRB Outriggers, currently under commission, expected to achieve sub-arcsecond localization for hundreds of FRBs per year [409]. In the longer term, upcoming radio telescopes with increased sensitivity will allow for better detection of faint, lensed FRBs [410–412]. Improving the coordination between FRB surveys and GW observatories will become vital for detecting lensed signals from both messengers.

#### (v) Studying the properties of and links between mergers and their hosts

There remain a great deal of unknowns about the host galaxies of GW mergers, and they remain an active field of study in astrophysics [413]. As only one GW event—the multi-messenger GW170817 detection—from all of O1–O3 has been confidently associated with a host galaxy, most studies about GWs and their host galaxies rely entirely on simulations of binary formation and galaxy evolution. However, lensing provides an opportunity to revolutionize the field of GW host population studies: in theory, as every lensed binary merger, bright or dark, has the capacity to be localized and its host identified, every lensed event could become a valuable contribution to studying the hosts of GWs.

Typically, GW binary formation is expected to correlate with certain properties of the host galaxy, such as mass, star formation rate and metallicity [413,414]. However, these conclusions are based largely on simulations combining stellar and galaxy evolution codes. Without lensing, studies of host galaxies are limited to GW170817-like detections, mergers confidently associated with AGN flares or exceptional BBHs sufficiently well-localized to be matched with a single galaxy [415]. With lensing, dark GW mergers have the possibility of being traced back to a singular host as discussed in §4c. And, as with GW170817, if a bright EM counterpart to a BNS is successfully observed, host identification becomes less challenging as a consequence of finding the BNS’s exact position, typically allowing for a confident identification of the host galaxy. However, the possible offset between the BNS/NSBH mergers and their host galaxies may make the association more challenging [416–418]. Thus, each lensed GW merger offers opportunities for multi-messenger host studies [16,18].

In addition to offering opportunities for direct host identification for binaries, lensing has the additional benefit that the mergers, and thus their hosts, can now originate from a variety of redshifts due to gravitational magnification enabling discoveries beyond the redshift frontier. This means that host population studies through lensing will inevitably unlock information about the redshift evolution of host populations through the Universe.

However, as mentioned before, merger host identification does not come without challenges, and it is likely that not all mergers can be directly identified with their hosts. GW merger hosts may be too dim to be observed, or the merger may be far enough offset from its host to leave host association uncertain. Even these cases provide their own valuable scientific applications. In the case of bright mergers, we can directly measure the offset between the merger and candidate hosts from optical imaging data [418], which can provide information about kicks at binary formation analogously to studies done on ‘hostless’ supernovae [419]. When the host is too dim for identification, we can also place constraints on the maximum luminosity—and therefore, mass—of the host galaxy for non-observation [57]. In the case of dark mergers, the host identification may be narrowed down to a few plausible candidate host systems that cannot be separated [16,243]. While this does not offer direct opportunities to study the host of the GW emitter, it still allows the study of limited candidates and places constraints on current assumptions used in simulations. Conversely, it is possible to use information from host-GW simulations to constrain further the list of candidates and possibly pin down the host once more in a method similar to [420].

It is therefore clear that multi-messenger studies of the hosts of lensed GW binaries will provide invaluable information towards understanding the properties of the hosts of compact binaries, understanding the evolution of this relationship with redshift, constraining the kicks created at binary formation, and likely a swathe of other avenues yet to be explored.

#### (vi) The physics of core-collapse supernovae

Currently, there are many competing models to describe the mechanics of core collapse of massive stars as they evolve into core-collapse supernovae (CCSNe) ([421] and references therein). Multi-messenger constraints on CCSNe from neutrinos and potentially GWs are therefore central to future progress in this field because alongside their well-known optical emission, CCSNe are responsible for a large fraction of detected long-duration GRBs [42,422–424] and the landmark detection of SN1987A confirmed them as sources of neutrinos [19–21]. Indeed, it is the ability of neutrinos and potential GW signals from CCSNe that are able to probe beneath the optically opaque envelope to constrain the precise timeline, geometry and thus physics of core collapse.

Multi-messenger gravitational lensing can enhance the study of core-collapse physics by taking advantage of the arrival time difference between magnified images of gravitationally lensed CCSN. In the optical, this can probe the early phase of the CCSNe light curve and can potentially constrain the size of the progenitor star just before core collapse [425]. Indeed, Rubin/LSST is forecast to detect many hundreds of gravitationally lensed CCSNe [53,54,56], offering great scope to ‘cherry pick’ optimal systems for detailed further study. For example, systems with a gravitationally lensed GRB counterpart and sufficient magnification to motivate pointed analysis of GW and neutrino datasets are likely to attract attention.

The main challenge that multi-messenger gravitational lensing faces in this science case is that the event rates may be very low. This is highlighted by the relatively small local volume within which contemporary/imminent neutrino and GW detectors are sensitive to signals from CCSNe (table 1). For example, pointed searches for GW signals associated with CCSNe are limited to those located within a distance of D<20Mpc [426]. This implies a small redshift horizon (§4b) and correspondingly low rate and large gravitational magnification. Further progress will therefore require investigating the potential synergy between the large number of gravitationally lensed CCSNe expected to be discovered by Rubin/LSST and the sensitivity of GW and neutrino detectors to only the most highly magnified events, including the development of robust strategies for selecting and confirming lensed CCSNe from the Rubin/LSST alert stream.

## Data Availability

This article has no additional data.

## Appendix A. Glossary

**Table:**

AGN	Active Galactic Nucleus
AT	Astronomical Telegram
ATLAS	Asteroid Terrestrial-impact Last Alert System
BH	Black hole
BBH	Binary Black Hole
BNS	Binary neutron star
CBC	Compact binary coalescence
CCSN	Core-collapse supernova
CDM	Cold dark matter
CE	Cosmic Explorer
CHIME	Canadian Hydrogen Intensity Mapping Experiment
CHORD	The Canadian Hydrogen Observatory and Radio-transient Detector
DM	Dark matter
EM	Electromagnetic
ET	Einstein Telescope
EoS	Equation of state
FRB	Fast radio burst
GBM	Gamma-ray burst monitor
GOTO	Gravitational Wave Optical Transient Observatory
GR	General relativity
GRB	Gamma-ray burst
GW	Gravitational wave
GWTC	Gravitational Wave Transient Catalog
$H_{0}$	Hubble constant
IMF	Initial mass function
iPTF	Intermediate Palomar Transient Factory
IR	Infrared
KAGRA	Kamioka Gravitational Wave Detector
LIGO	Laser Interferometer Gravitational-Wave Observatory
LSST	Legacy Survey of Space and Time
LS4	La Silla Schmidt Southern Survey
LVK	LIGO-Virgo-KAGRA
MSD	Mass sheet degeneracy
NS	Neutron star
NSBH	Neutron star-black hole
PanSTARRS	Panoramic Survey Telescope and Rapid Response System
PBH	Primordial black hole
Rubin	Vera C. Rubin Observatory
SDSS	Sloan Digital Sky Survey
SKA	Square kilometre array
SFRD	Star formation rate density
SN	Supernova
SNIa	Type Ia supernova
SNR	Signal-to-noise ratio
SLSN	Super-luminous supernova
SVOM	Space variable objects monitor
ToO	Target of opportunity
TDE	Tidal disruption event
UV	Ultraviolet
WFD	Wide Fast Deep survey
XG	Next-generation GW detectors
ZTF	Zwicky transient facility
